# Transfer RNA gene arrangement and codon usage in vertebrate mitochondrial genomes: a new insight into gene order conservation

**DOI:** 10.1186/1471-2164-11-479

**Published:** 2010-08-19

**Authors:** Takashi P Satoh, Yukuto Sato, Naoharu Masuyama, Masaki Miya, Mutsumi Nishida

**Affiliations:** 1 Collection Center, National Museum of Nature and Science, 3-23-1 Hyakunincho, Shinjuku-ku, Tokyo 169-0073, Japan; 2 Division of Population Genetics, National Institute of Genetics, 1111 Yata, Mishima, Shizuoka 411-8540, Japan; 3 Department of Marine Bioscience, Atmosphere and Ocean Research Institute, The University of Tokyo, 5-1-5 Kashiwanoha, Kashiwa-shi, Chiba 277-8564, Japan; 4 Resonarch Co., Ltd., 2-2-23 Josui-shinmachi, Kodaira, Tokyo 187-0023, Japan; 5 Department of Zoology, Natural History Museum and Institute, 955-2 Aoba-cho, Chuo-ku, Chiba 260-8682, Japan

## Abstract

**Background:**

Mitochondrial (mt) gene arrangement has been highly conserved among vertebrates from jawless fishes to mammals for more than 500 million years. It remains unclear, however, whether such long-term persistence is a consequence of some constraints on the gene order.

**Results:**

Based on the analysis of codon usage and tRNA gene positions, we suggest that tRNA gene order of the typical vertebrate mt-genomes may be important for their translational efficiency. The vertebrate mt-genome encodes 2 rRNA, 22 tRNA, and 13 transmembrane proteins consisting mainly of hydrophobic domains. We found that the tRNA genes specifying the hydrophobic residues were positioned close to the control region (CR), where the transcription efficiency is estimated to be relatively high. Using 47 vertebrate mt-genome sequences representing jawless fishes to mammals, we further found a correlation between codon usage and tRNA gene positions, implying that highly-used tRNA genes are located close to the CR. In addition, an analysis considering the asymmetric nature of mtDNA replication suggested that the tRNA loci that remain in single-strand for a longer time tend to have more guanine and thymine not suffering deamination mutations in their anticodon sites.

**Conclusions:**

Our analyses imply the existence of translational constraint acting on the vertebrate mt-gene arrangement. Such translational constraint, together with the deamination-related constraint, may have contributed to long-term maintenance of gene order.

## Background

The animal mitochondrial (mt)-genome generally encodes 13 protein, 2 rRNA, and 22 tRNA genes. Although their arrangement is rather variable among invertebrate mt-genomes, a typical gene arrangement has been highly conserved among vertebrate mt-genomes from jawless fishes to mammals with some exceptions [1,2]. This implies an extremely long-term persistence of mt-gene order probably for > 500 million years across diverse clades of vertebrates. However, it has been unclear whether such high-conservation of gene order is a consequence of some constraints, or whether it results only by sharing a common ancestry. This has been a long-standing enigma for more than 20 years since the initial reports of the whole mt-genome sequence of vertebrates [3].

To address this problem, we analyzed codon usage and tRNA gene arrangements of the vertebrate mt-genomes to examine possible constraints on the gene order of vertebrate mt-genomes.

## Results and Discussion

### Amino acid usage and tRNA gene arrangement

**Figure 1:**
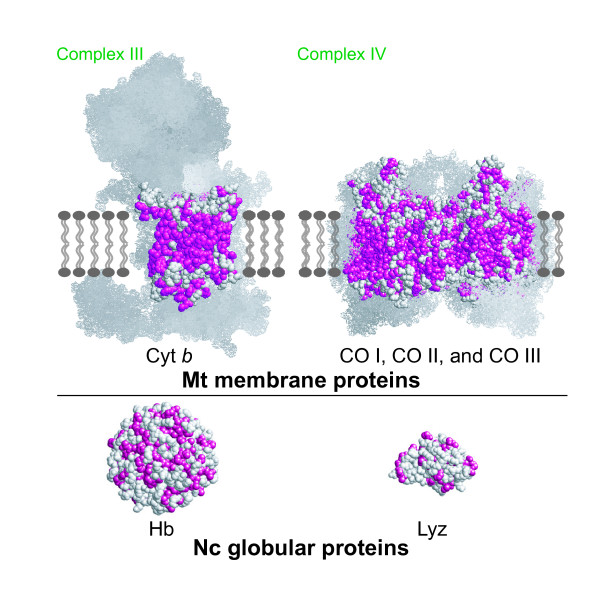
**Spatial distribution of hydrophobic residues in the representative proteins encoded by vertebrate mitochondrial (mt) and nuclear (nc) genomes**. Hydrophobic residues (Phe, Val, Leu, Ile, Met, Trp, Ala, Gly, and Pro) are colored magenta. The upper panels show transmembrane proteins encoded by mt-genomes: bovine cytochrome *bc *complex (complex III) (left; Protein Data Bank ID [PDB]: 1SQQ) and cytochrome *c *oxidase complex (complex IV) (light; PDB: 1V55). In these panels, the protein subunits encoded by mt-genes are shown by space-filling models, and the remaining subunits encoded by nc genes are shown by Van der Waals' surface dot models. The lower panels show globular proteins encoded by nc-genomes: human hemoglobin A (PDB: 1BZ0) and lysozyme (PDB: 133L). The 3 D graphical models were generated and processed using the program RasMol [30].

**Figure 2:**
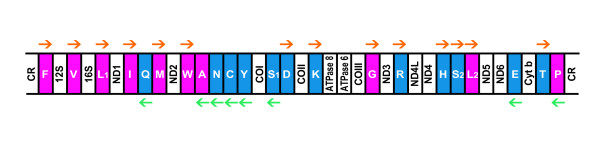
**Schematic diagram outlining the typical gene order in the vertebrate mt-genome**. The tRNA genes (designated using single-letter amino acid codes) that specify hydrophobic and hydrophilic amino acids are colored magenta and blue, respectively. Orange and green arrows show the transcriptional orientation of each tRNA gene on the heavy (H)-strand and light (L)-strand, respectively. CR, the control region; 12 S and 16 S, the 12 S and 16 S ribosomal RNA genes; ND1-6, and 4L, NADH dehydrogenase subunits 1-6 and the 4L gene; COI-III, cytochrome *c *oxidase subunits I-III genes; ATPase 6 and 8, ATPase subunits 6 and 8 genes; Cyt *b*, the cytochrome *b *gene; L_1 _and L_2 _indicate the tRNA-Leu (UUR) and tRNA-Leu (CUN) genes, respectively; S_1 _and S_2_, the tRNA-Ser (UCN) and tRNA-Ser (AGY) genes.

We began by focusing on the fact that all of the 13 proteins encoded by the vertebrate mt-genomes are transmembrane proteins [3], which are rich in hydrophobic amino acid residues (Fig. 1). The frequency of codon usage for hydrophobic amino acids is, consequently, higher in the vertebrate mt-genome (0.624, the mtREV matrix [4]) compared to a general nuclear genome (0.490, the JTT matrix [5]). Based on this observation, we divided the mt-tRNA genes into two groups according to the hydrophobicity of their corresponding amino acids, and then examined their positions in the vertebrate mt-genomes (Fig. 2). This revealed that genes for tRNAs that specify hydrophobic amino acids (Fig. 2, colored magenta) are located close to the control region (CR; [6]) compared with the other genes for tRNAs specifying hydrophilic amino acids (Fig. 2, colored blue; *p *= 0.0295, Mann-Whitney *U*-test, *U *= 27.000, *n*_1 _= 10, *n*_2 _= 12; the detailed data based on human mt-genome is shown in Table 1).

**Table 1:** Comparison of the positions of mitochondrial (mt) tRNA genes corresponding to hydrophobic and hydrophilic amino acids based on human mt-genome data

**Hydrophobic group**			**Hydrophilic group**		
	
**tRNA**	**Strand**^**a**^	**Distance**^**b**^	**tRNA**	**Strand**^**a**^	**Distance**^**b**^
	
Phe	H	1	Glu	L	1282
Pro	L	1	Asp	H	6941
Val	H	1026	Lys	H	7718
Leu (UUR)	H	2653	Ser (UCN)	L	8508
Ile	H	3686	Arg	H	9828
Met	H	3825	Tyr	L	10133
Trp	H	4935	Cys	L	10198
Gly	H	9414	Asn	L	10295
Ala	L	10369	His	H	11561
Leu (CUN)	H	11689	Gln	L	11624
			Ser (AGY)	H	11630
			Thr	H	15311

^a ^The mtDNA strand on which the tRNA gene occurs: H, heavy strand; L, light strand.

^b ^Base-pair distance between the 3'-end of control region and the 5'-end of each tRNA gene.

Such tRNA gene localization may indicate that, in the typical vertebrate mt-genomes, highly-used tRNA genes are located in the genomic region close to the CR, where the transcription efficiency is thought to be relatively high. The transcription of the vertebrate mt-genome is initiated from regulatory elements within the CR [6,7], and thus, the complete transcription of the genes into mRNA and functional RNAs would be more successful in the genomic region closer to the CR. In fact, the two rRNA genes immediately adjacent to the CR (12 S and 16S; see Fig. 2) are highly expressed [8]. Likewise, the tRNA genes localized close to the CR, which specify hydrophobic residues, would also be highly expressed. Such an efficient production of the highly-used tRNAs may be favorable for translation of vertebrate mt-genomes.

### Correlation between codon usage and tRNA gene position

Given the increasing availability of full-length mt-genome sequence data from a broad range of vertebrate species, the tRNA gene arrangement with regards to amino acid usage can be assessed more quantitatively by analyzing a correlation between physical (base pair [bp]) distance from the CR to each tRNA gene and codon usage of the mt-genome. We analyzed correlation between these two parameters using both parametric and non-parametric methods based on the mt-genome sequences of 47 representative vertebrates from jawless fish, cartilaginous fish, ray-finned fish, amphibians, reptiles, birds, and mammals (species names are listed in Table 2). Among these 47 species, 33 represent the "evolutionarily stable" mt-gene orders including the typical gene arrangement of vertebrates and slightly rearranged gene orders of lamprey and birds (supplementary Fig. S1 [see Additional file 1]). The remaining 14 species from ray-finned fish, amphibians, and reptiles represent rearranged gene orders within these lower taxa. More rearranged mt-genomes were sampled from ray-finned fish (8 species) than from amphibians and reptiles (6 species) because ray-finned fish is a sister group to all tetrapods, and shows greater diversity in terms of mt-gene rearrangements.

**Table 2:** List of the species studied and the DDBJ/EMBL/GenBank accession numbers of their mitochondrial genome sequences

Scientific name	Common name	**Accession No**.
**Evolutionarily stable gene orders**		
Agnatha		
*Eptatretus burgeri*	Inshore hagfish	AJ278504
*Myxine glutinosa*	Atlantic hagfish	AJ404477
*Petromyzon marinus*	Sea lamprey	U11880
		
Chondrichthyes		
*Chimaera monstrosa*	Rabbitfish	AJ310140
*Scyliorhinus canicula*	Catshark	Y16067
*Squalus acanthias*	Spiny dogfish	Y18134
*Heterodontus francisci*	Horn shark	AJ310141
*Raja radiata*	Thorny skate	AF106038
		
Actinopterygii		
*Lepisosteus spatula*	Alligator gar	AP004355
*Cyprinus carpio*	Common carp	AP009047
*Gadus morhua*	Atlantic cod	X99772
*Pagrus major*	Seabream	AP002949
*Paralichthys olivaceus*	Bastard halibut	AB028664
		
Amphibia		
*Ambystoma mexicanum*	Axolotl	AJ584639
*Andrias japonicus*	Giant salamander	AB208679
*Bombina bombina*	Fire-bellied toad	AY458591
*Xenopus laevis*	Clawed frog	M10217
*Ichthyophis glutinosus*	Caecilian	AY456251
		
Reptilia		
*Chelonia mydas*	Seaturtle	AB012104
*Geochelone pardalis*	Tortoise	DQ080041
*Gekko gecko*	Tokay	AY282753
*Iguana iguana*	Iguana	AJ278511
*Geocalamus acutus*	Worm lizard	AY605476
		
Aves		
*Gallus gallus*	Chicken	X52392
*Nipponia nippon*	Crested ibis	AB104902
*Vidua chalybeata*	Widowfinch	AF090341
*Falco peregrinus*	Peregrine falcon	AF090338
*Buteo buteo*	Buzzard	AF380305
		
Mammalia		
*Homo sapiens*	Human	AF347015
*Mus musculus*	Mouse	AY172335
*Bos taurus*	Cattle	AY526085
*Canis lupus*	Gray wolf	DQ480505
*Balaenoptera musculus*	Blue whale	X72204
		
**Rearranged gene orders within lower taxa**		
Actinopterygii		
*Eurypharynx pelecanoides*	Pelican eel	AB046473
*Saccopharynx lavenbergi*	Gulper eel	AB047825
*Gonostoma gracile*	Slender fangjaw	AB016274
*Myctophum affine*	Lantern fish	AP002922
*Caelorinchus kishinouyei*	Grenadier	AP002929
*Aspasma minima*	Clingfish	AP004453
*Aulostomus chinensis*	Trumpet fish	AP009197
*Chlorurus sordidus*	Parrot fish	AP006567
		
Amphibia		
*Rana nigromaculata*	Pond frog	AB043889
*Buergeria buergeri*	Kajika frog	AB127977
*Rhacophorus schlegelii*	Tree frog	AB202078
		
Reptilia		
*Boa constrictor*	Boa	AB177354
*Dinodon semicarinatus*	Colubrid snake	AB008539
*Gloydius blomhoffii*	Pit viper	EU913477

In the correlation analysis, we aimed to eliminate the effects of shared common ancestry [9] based on independent contrast analysis [10] of the codon usage and tRNA positions. Independent contrasts for these two variables were estimated using the program CAIC [11] based on a composite tree of the sampled species that was constructed from recent molecular phylogenies for the major clades of vertebrates (supplementary Fig. S2 [see Additional file 1]). This method focuses on differences only between sister lineages or nodes in a phylogeny, which have arisen after a split, therefore yielding sets of independent "contrasts" [10]. Our data points were derived from ancestral states (namely, various nodes across the tree) based on independent contrasts of tRNA genes (listed in supplementary Table S1, Table S2, and Table S3 [see Additional file 1]). Thus, many data points were above the level of major clades such as mammals, birds, reptiles, amphibians, actinopterygians, chondrichthyans, and agnathans, and the total number of data points became much smaller than the number of pair-wise comparisons.

**Figure 3:**
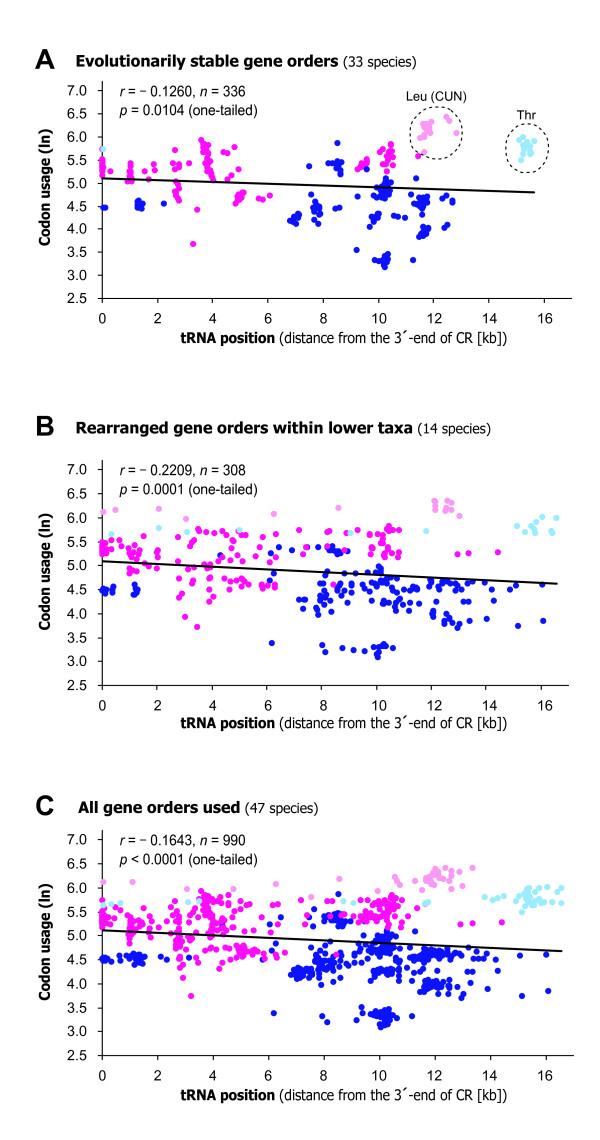
**A linear regression plot between position of tRNA genes and usage of the corresponding codon**. The data points were derived from an independent contrasts analysis using the program CAIC [11] based on vertebrate mt-genome sequences from (**A**) 33 species with evolutionarily stable gene order, (**B**) 14 species with rearranged gene orders within lower taxa, and (**C**) all of the 47 species selected (Table 2) and recent molecular phylogenies for vertebrates (supplementary Fig. S2 [see Additional file 1]). Data points for the tRNA genes that specify hydrophobic and hydrophilic amino acids are colored magenta and blue, respectively; exceptionally, the data points for the tRNA-Leu (CUN) and tRNA-Thr are colored light pink and light blue, respectively. The regression lines were derived from the all data points in each plot. When the tRNA genes of H-strand and L-strand were analyzed separately, the correlation remained negative for either of the strands (data not shown).

As a result, we found a significant correlation between the tRNA positions and codon usage (Fig. 3; evolutionarily stable gene orders: Pearson's correlation coefficient *r *= -0.1260, one-tailed *p *= 0.0104, *n *= 336; Spearman rank-correlation coefficient *r*_s _= -0.0827, *t *= -2.2171, one-tailed *p *= 0.0136, *n *= 336, d.f. = 334; rearranged gene orders within lower taxa: Pearson's correlation coefficient *r *= -0.2209, one-tailed *p *= 0.0001, *n *= 308; Spearman rank-correlation coefficient *r*_s _= -0.2336, *t *= -3.9727, one-tailed *p *< 0.0001, *n *= 308, d.f. = 306; all of the 47 mt-genomes: Pearson's correlation coefficient *r *= -0.1643, one-tailed *p *< 0.0001, *n *= 990; Spearman rank-correlation coefficient *r*_s _= -0.1441, *t *= -4.4827, one-tailed *p *< 0.0001, *n *= 990, d.f. = 988). These results imply that the tRNA genes that correspond to highly-used codons were located nearer the CR compared with the others. The data points for the tRNA-Leu (CUN) and tRNA-Thr genes, however, deviated visibly from the trend. The usage of their corresponding codons was relatively high, although their genomic positions were distant from their putative transcription start site in the CR (see Fig. 3). When these two genes were excluded, the correlation became stronger (not shown as figures; evolutionarily stable gene orders: *r *= -0.4207, one-tailed *p *< 0.0001, *n *= 304; rearranged gene orders within lower taxa: *r *= -0.2760, one-tailed *p *< 0.0001, *n *= 294; all of the 47 mt-genomes: *r *= -0.4121, one-tailed *p *< 0.0001, *n *= 900).

The significance of the above correlations, however, may be due to the larger number of degrees of freedom generated by multiple-species comparisons on multiple tRNA genes, although we sought to eliminate the effect of shared common ancestry as described above. To limit this effect and to corroborate our results, we took two other approaches: first, we averaged codon usage and distance from the CR for each of 22 tRNA across taxa, respectively, yielding 22 independent data points of 22 non-homologous tRNA genes. This set of data might reflect an ancient adaptation between codon usage and tRNA positions of an original vertebrate mt-genome. Second, we analyzed variations of codon usage and distance from the CR of each of the 22 tRNA genes across taxa, specifically focusing on the rearranged gene orders within lower taxa. The second analysis might detect a recent adaptation between codon usage and tRNA positions in taxonomic groups concerned (lower than an order level; for details, see supplementary Fig. S2 [see Additional file 1]).

**Figure 4:**
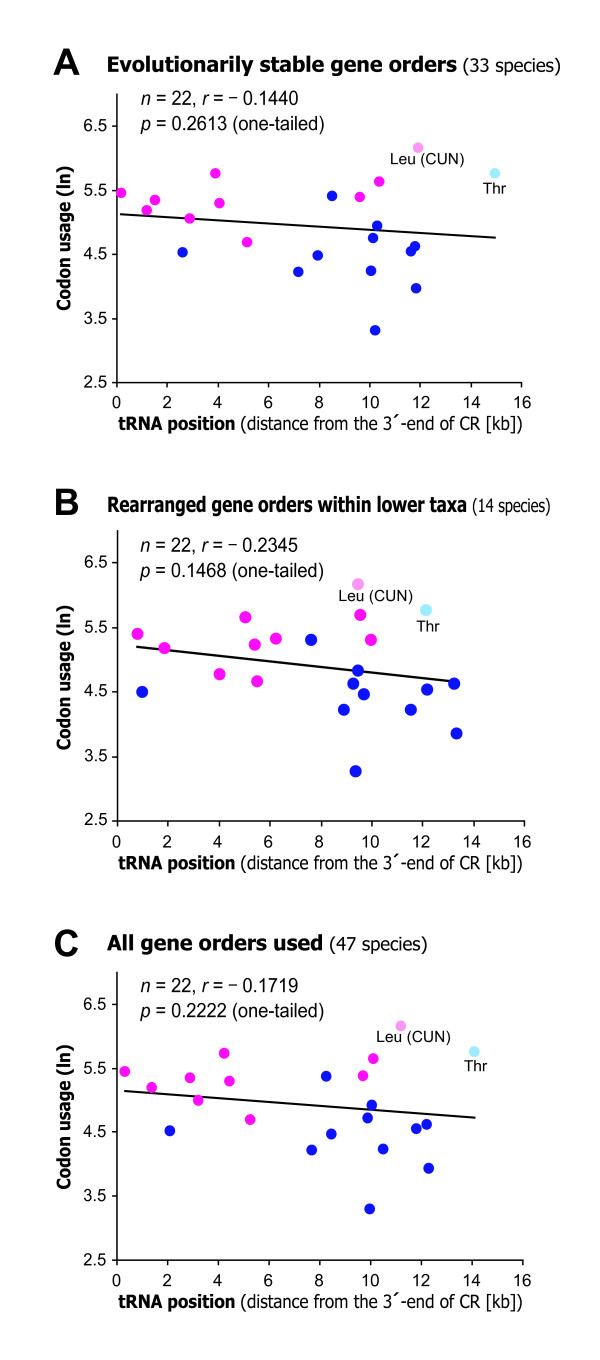
**A linear regression plot between mean position of the tRNA genes and mean usage of the corresponding codon**. The data points represent the average of each of tRNA across the mt-genome sequences from (**A**) 33 species with evolutionarily stable gene order, (**B**) 14 species with rearranged gene orders within lower taxa, and (**C**) all of the 47 species selected (Table 2). Data points for the tRNA genes that specify hydrophobic and hydrophilic amino acids are colored magenta and blue, respectively; exceptionally, the data points for the tRNA-Leu (CUN) and tRNA-Thr are colored light pink and light blue, respectively. The regression lines were derived from the all data points in the plot.

By way of the first approach, we found a weak (but statistically not significant) correlation between mean codon usage and tRNA positions (Fig. 4; evolutionarily stable gene orders: *r *= -0.1440, one-tailed *p *= 0.2613, *n *= 22; rearranged gene orders within lower taxa: *r *= -0.2345, one-tailed *p *= 0.1468, *n *= 22; all of the 47 mt-genomes: *r *= -0.1719, one-tailed *p *= 0.2222, *n *= 22), implying that the tRNA genes corresponding to highly-used codons were located nearer the CR. This relationship became stronger and the correlation significant when the outlier tRNA-Leu (CUN) and tRNA-Thr genes were excluded (not shown as figures; evolutionarily stable gene orders: *r *= -0.4510, one-tailed *p *= 0.0230, *n *= 20; rearranged gene orders within lower taxa: *r *= -0.4206, one-tailed *p *= 0.0324, *n *= 20; all of the 47 mt-genomes: *r *= -0.4503, one-tailed *p *= 0.0232, *n *= 20). These results would support the correspondence between codon usage and mt-tRNA positions possibly in the origin of the vertebrate mt-genome gene order, although such constraint might have been weak.

The second approach involving the meta-analysis of the results of the respective 22 tRNA gene sets showed that the rearranged gene orders within lower taxa had little effect on the preexisting correlation between codon usage and tRNA positions (an overall weighted Fisher's *r *= 0.0722, Stouffer's combined *p *= 0.3128; the results of the correlation in the respective 22 tRNAs are shown in the supplementary Fig. S3 and Fig. S4 [see Additional file 1]). This result can be explained as a result of elimination of novel gene arrangement deviating from the relationship. In fact, among 13 of phylogenetically independent cases of gene-order rearrangements (supplementary Fig. S5 [see Additional file 1]), three cases (deep-sea eels, frogs, and Tuatara) were observed to have improved correlation between tRNA position and codon usage compared to the evolutionarily stable gene orders; majority of the cases of gene order rearrangements showed no improvement in the relationship (detailed data are shown in supplementary Table S4 [see Additional file 1]). This implies that not a few gene order rearrangements without improvement have existed for some periods of evolutionary time. It is noted, however, that the correlation between tRNA position and codon usage was significant (or marginally significant) for the above three cases only. Their novel gene order arrangements might be maintained through some forms of natural selection.

On the basis of these sets of analyses, we propose that the tRNA gene arrangement of vertebrate mt-genomes, and possibly that of an ancestral, original vertebrate mt-genome, may be adaptive with regard to translational efficiency. The genes close to the CR, where the transcription initiation sites of both strands exist, appear to be highly expressed in the vertebrate mt-genomes [8]. Consequently, genes of tRNAs specifying highly-used codons would be favorably located close to the CR to ensure the efficient translation of the protein-coding genes in the vertebrate mt-genomes.

### Mitochondrial gene arrangement and translational constraint

On the basis of the results obtained from our analyses, we suggest the existence of translational constraint on the positions of mt-tRNA genes, but not on their gene copy numbers, in the vertebrate mt-genomes, although the constraint may be weak. In nuclear genomes, translational selection is known to promote adaptation of tRNA gene number to the usage of the corresponding codon [12,13]. Clear association of tRNA gene number with codon usage has been observed in the genomes of various organisms ranging from *E. coli *to humans [14-19]. The vertebrate mt-genome is also likely exposed to translational selection because vertebrates are considered to be metabolically active and have high rates of ATP synthesis. However, translational selection would not act at the level of tRNA gene numbers in the vertebrate mt-genome since it is extremely compact and the number of contained genes is limited.

Recently, some studies suggest the replication and translational constraints affected the positions of translational genes such as RNA polymerase, rRNA, and tRNA genes in bacterial genomes [20], and abundant and broadly expressed genes in the human genome [21]. Such constraints associated with translation and gene expression may also have limited the gene order rearrangement of vertebrate mt-genomes, specifically the rearrangements which interfere with transcriptional efficiency of mt-tRNAs (see Fig. 3B, Fig. 4B, and supplementary Fig. S3 and Fig. S4 [see Additional file 1]). This constraint may have driven the conservation of the mt-gene arrangement among vertebrates from jawless fishes to mammals for more than 500 million years.

Gene-order rearrangements are often found in vertebrate mt-genomes within lower taxonomic categories such as families, genera, and species (166/769 = 21.6% of species [22]), however, there are no extensive rearrangements shared across higher taxa, which are likely to have persisted for long evolutionary periods of time [1]. This observation further implies the existence of constraint on vertebrate mt-gene orders, possibly through translational efficiency as discussed above. Exceptionally, mt-genomes of birds and lampreys show some little deviation from the typical gene order; either of the bird or lamprey have mt-genomes showing some changes in tRNA-Glu, tRNA-Thr, and tRNA-Pro gene positions (see supplementary Fig. S1 [see Additional file 1]). These non-typical gene orderings, however, interfere little with the correlation reported above (when birds and lampreys were excluded; *r *= -0.1499, one-tailed *p *= 0.0071, *n *= 267). This also implies that the deviations of the tRNA-Leu (CUN) and tRNA-Thr from the supported relationship (see Fig. 3) do not arise from the rearrangements of birds and lampreys.

**Figure 5:**
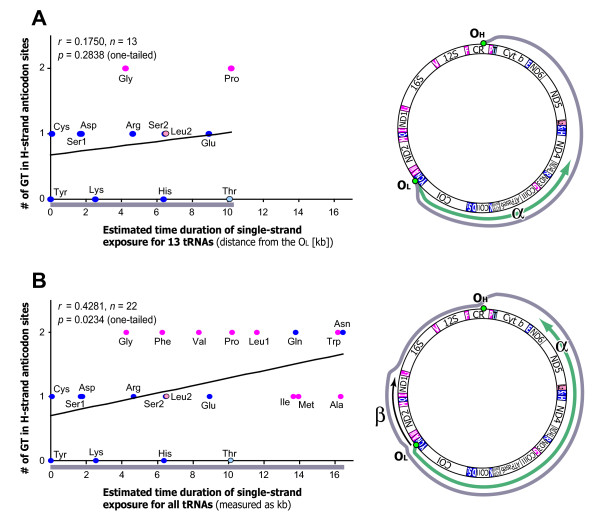
**Correlation between the number of guanine (G) and thymine (T) in 1st and 2nd anticodon positions of tRNA loci and expected time duration of single-strand exposure during mtDNA replication**. The analysis is based on a human mt-genome. Data points for the tRNA genes that specify hydrophobic and hydrophilic amino acids are colored magenta and blue, respectively; exceptionally, tRNA-Leu (CUN) and tRNA-Thr are shown in light pink and light blue, respectively. (**A**) Analysis for tRNA genes located between the O_L _and O_H _along the direction of L-strand replication (tRNA-Cys, -Tyr, -Ser1, -Asp, -Lys, -Gly, -Arg, -His, -Ser2, -Leu2, -Glu, -Thr, and -Pro). The expected time duration of single-strand exposure was measured as distance from the O_L _along the direction of L-strand replication. This distance is named as α, as depicted in the right part of the panel A. (**B**) Analysis for all tRNA genes. For the above-mentioned 13 tRNA genes (described in the legend of the panel A), the duration of single-strand exposure was measured as α. For the other 9 tRNA genes (tRNA-Asn, -Ala, -Trp, -Met, -Gln, -Ile, -Leu1, -Val, and -Phe), the duration of single-strand exposure was estimated as (α - β), where β is distance from the O_L _along the opposite direction of L-strand replication. This is because, among those 9 tRNA loci, the tRNAs located more distant from the O_L _along the opposite direction of L-strand replication remain in double-stranded DNA for a longer time based on the displacement model of mtDNA replication [26].

We found two tRNA genes that contradict our notion. The tRNA-Leu (CUN) and tRNA-Thr genes were located distant from their putative transcription start site in the CR, but the usage of their corresponding codons was relatively high (Fig. 3 and Fig. 4). The presence of such outlier tRNAs is not strange because mt-tRNA genes are also under constraints associated with their other functions such as punctuation markers during the pre-mRNA processing of adjacent genes [7]. However, one attractive possible constraint on mt-tRNA position is deamination gradients during replication and possibly transcription of the mt-DNA [23]. Deamination commonly occurs in single-stranded DNA exposed during replication or transcription, causing mutations from adenine to guanine and from cytosine to thymine [23,24]. Consequently, the gene loci exposed in single strand for longer time in replication and transcription are more prone to suffer these deamination mutations. In the replication of mtDNA [25,26], the Heavy (H)-strand is initially replicated from the H-strand replication origin (O_H_) in the CR. The parental, original H-strand is exposed as a single strand (see right part of the Fig. 5A, gray line). Subsequently, using this original H-strand as a lagging strand, the Light (L)-strand is replicated from the L-strand replication origin (O_L_) in a WANCY region [26] (Fig. 5A, green arrow). Based on this view, the tRNA genes located more distant from the O_L _along the direction of L-strand replication would be exposed as a single strand for a longer time. Consequently, such tRNA genes are more likely to undergo the deamination mutations. A similar deamination gradient might also exist in transcription initiated from the CR [23].

The present analysis considering the asymmetric nature of mtDNA replication provided results match with the above prediction (Fig. 5). The tRNA loci that are expected to be exposed as a single strand for a longer time tend to have more guanine (G) and thymine (T) in their anticodon region on the coding strands (Fig. 5A: tRNA loci located between the O_L _and O_H _along the direction of L-strand replication: *r *= 0.1750 one-tailed *p *= 0.2838, *n *= 13; Fig. 5B: all tRNA genes: *r *= 0.4281 one-tailed *p *= 0.0234, *n *= 22). This suggests that the tRNA gene arrangement of typical vertebrate mt-genome is also adaptive in avoiding mutations in anticodons through deamination during replication, and possibly, in transcription [23]. Regarding the correlation coefficients, the deamination-related constraint may be stronger than the codon/amino-acid usage-related constraint discussed above.

## Conclusion

In this paper, we propose that the high conservation of the gene arrangement of the vertebrate mt-genome is underpinned not only by a shared common ancestry, but also by translational constraint acting on the tRNA gene arrangement. This conclusion can be derived from the simple observation that the mt-tRNA genes corresponding to hydrophobic amino acids, which are frequently used in translation of the mt-genes, are localized close to the CR. In addition, an analysis considering the asymmetric nature of mtDNA replication suggested that deamination-related constraint against mutations in tRNA anticodons is also an important determinant of the tRNA gene arrangement in the typical vertebrate mt-genome. The translational constraint together with the deamination-related constraint may have contributed to shaping and maintaining the typical gene order of the vertebrate mt-genomes.

## Methods

### Taxonomic sampling of mitochondrial genome data

To consider variation in codon usage and gene arrangement across typical vertebrate mt-genomes, we chose five species from each of mammals, birds, reptiles, amphibians, actinopterygians, and chondrichthyans, and three species from agnathans, for which only few mt-genome sequences were available in databases. Those 33 mt-genomes are defined as "evolutionarily stable" gene orders. In addition, to include mt-genomes that have rearranged gene orders within lower taxa, we chose eight species from actinopterygians and three species from reptiles and amphibians, respectively. Those 14 mt-genomes are defined as "rearranged gene orders within lower taxa". Species names and GenBank accession numbers of the mt-genomes are listed in Table 2: these species were selected to represent a broad niche breadth. The invertebrates could not be analyzed in this study, because a transcription system of the mt-genome and a sound phylogenetic framework are unclear for most of them.

### Measuring codon usage and the position of each tRNA gene

The usage of each codon was counted in the sequence of the 13 protein-coding genes (ND1, ND2, ND3, ND4, ND4L, ND5, ND6, CO I, CO II, CO III, ATPase6, ATPase8, and Cyt *b*) of the mt-genomes examined. The overlapping codons between ATPase 8 and ATPase 6, and between ND4L and ND4 were considered once for each gene, because the open reading frame was different among these neighboring genes. To measure the position of each tRNA gene, base-pair distances from the 3' end of CR to the 5' end of each tRNA gene were counted in their respective positions on the H- and L-strands of mt-genome sequences. Although the accurate locations of the transcription start sites of mt-genome are unknown in most of the vertebrate species, it is assumed that the transcription start site for heavy and light strands may differ in distance from the 3' end of CR on the respective strand. Therefore, we examined whether such supposed differences affect the analysis in this study, and we found that the hypothetical differences of ± 150 bp and ± 500 bp in distance, which are based on a reference [27], do not affected the significance of the results of Mann-Whitney *U*-test and correlation analyses shown in the Results. Thus, we considered that measuring the positions of tRNA genes based on their base pair distances from the 3' end of the CR is justified.

### Regression analysis considering the effects of shared common ancestry

To examine whether the frequency of usage of each codon varies with the position of its corresponding tRNA gene (the base-pair distance from the CR), we calculated Pearson's correlation coefficient (*r*) and Spearman rank-correlation coefficient (*r*_s_), and evaluated the significance of the relationship both parametrically and non-parametrically, respectively. To account for the effect of shared common ancestry [9], "independent contrasts" [10] for these two variables were estimated using the program CAIC [11] based on a composite tree of the sampled species (supplementary Fig. S2 [see Additional file 1]). The typical mt-gene order has predominated and persisted in most of the major vertebrate lineages for more than 500 million years, however, local gene order rearrangements and codon usage variation have been observed and described in vertebrates [1,2,28]. By considering such potential changeability of mt-gene order and codon usage, we regarded the data points obtained from independent contrast analysis as virtually independent of each other, although all vertebrate mt-genomes share a common ancestor. The analysis using the program CAIC was performed using logarithmically transformed data to focus on the proportional change in the variables. The validity of this approach is discussed in the CAIC User's Guide [29].

## Abbreviations

BP: base-pair; CR: control region; MT: mitochondria; TRNA: transfer RNA.

## Authors' contributions

TPS, YS, and MN designed the study. TPS, YS, and NM carried out the analyses. TPS and YS drafted the manuscript. MM and MN participated in coordination and helped to draft the manuscript. All authors read and approved the final version of the manuscript.

## Supplementary Material

Additional file 1 **Supplementary figures and tables**. This PDF file includes supplementary figures S1--S5 and tables S1--S4. Click here for file
