# Supplementary material for: Transfer RNA gene arrangement and codon usage in vertebrate mitochondrial genomes: a new insight into gene order conservation
Source: BMC Genomics. 2010 Aug 19;11:479. doi: 10.1186/1471-2164-11-479 (PMC2996975; doi:10.1186/1471-2164-11-479)
Supplement: Additional file 1 — Supplementary figures and tables. This PDF file includes supplementary figures S1--S5 and tables S1--S4. [file 1471-2164-11-479-S1.PDF]

Supplementary figure legends

Figure S1 - Schematic diagram outlining the gene order in mitochondrial genome (mt-genome) that represents each of the major groups of vertebrates. The tRNA genes (designated using single-letter amino acid codes) that specify hydrophobic and hydrophilic amino acids are colored magenta and blue, respectively. Arrows show the transcriptional orientation of the tRNA genes. Green bars show gene arrangements that deviate from the typical (conserved) gene order, but are shared by higher taxa (e.g., lamprey and birds; indicated by star symbol shown in the right side of the figure). L_1 and L_2 indicate the tRNA-Leu (UUR) and tRNA-Leu (CUN) genes, respectively; S_1 and S_2, the tRNA-Ser (UCN) and tRNA-Ser (AGY) genes; 12S and 16S, the 12S and 16S ribosomal RNA genes; ND1-6, and 4L, NADH dehydrogenase subunits 1-6 and the 4L gene; COI-III, cytochrome *c* oxidase subunits I–III genes; ATPase 6 and 8, ATPase subunits 6 and 8 genes; Cyt *b*, the cytochrome *b* gene; CR, the control region.

Figure S2 - Composite phylogenetic tree of the 47 species examined. The mitochondrial genomes of most vertebrates have the “typical gene order”, whereas those of birds and sea lamprey deviate from the typical gene order (denoted by star-mark; see supplementary Fig. S1). In addition, some species in amphibians, reptiles, and actinopterygians (indicated by bold face letters) have “rearranged gene orders within lower taxa”, which is not shared by all species of the higher taxonomic group (e.g., amphibians or reptiles). The papers cited in the phylogenic tree are shown below.

Figure S3 - A linear regression plot between position and usage of the corresponding codon of each tRNA gene (1). The data points were based on vertebrate mt-genome

sequences of (**A**) evolutionarily stable gene order from 33 species, and (**B**) all of the 47 species selected including 14 species that have rearranged gene orders (see Table 2 in the main text). Data points for the tRNA genes that specify hydrophobic and hydrophilic amino acids are colored red and blue, respectively. The regression lines were derived from the all data points in each plot.

Figure S4 - A linear regression plot between position and usage of the corresponding codon of each tRNA gene (2). The data points were based on vertebrate mt-genome sequences of (**A**) evolutionarily stable gene order from 33 species, and (**B**) all of the 47 species selected including 14 species that have rearranged gene orders (see Table 2 in the main text). Data points for the tRNA genes that specify hydrophobic and hydrophilic amino acids are colored red and blue, respectively; exceptionally, the data points for the tRNA-Leu (CUN) and tRNA-Thr are colored light pink and light blue, respectively. The regression lines were derived from the all data points in each plot.

Figure S5 - Major events of mitochondrial gene-order rearrangements mapped on the phylogenetic tree of vertebrates studied. Species names in bold letters indicate species that have a mitochondrial genome with "rearranged gene orders within lower taxa". Circles on the tree denote supposed events of phylogenetically independent gene-order rearrangement. Magenta and blue arrows indicate species that have stronger and opposite relationships, respectively, between tRNA position and codon usage compared to the evolutionarily-stable gene orders. Green rectangles indicate species that have the relationship moderately similar to the evolutionarily-stable gene orders. The detailed data are shown in supplementary Table S4.

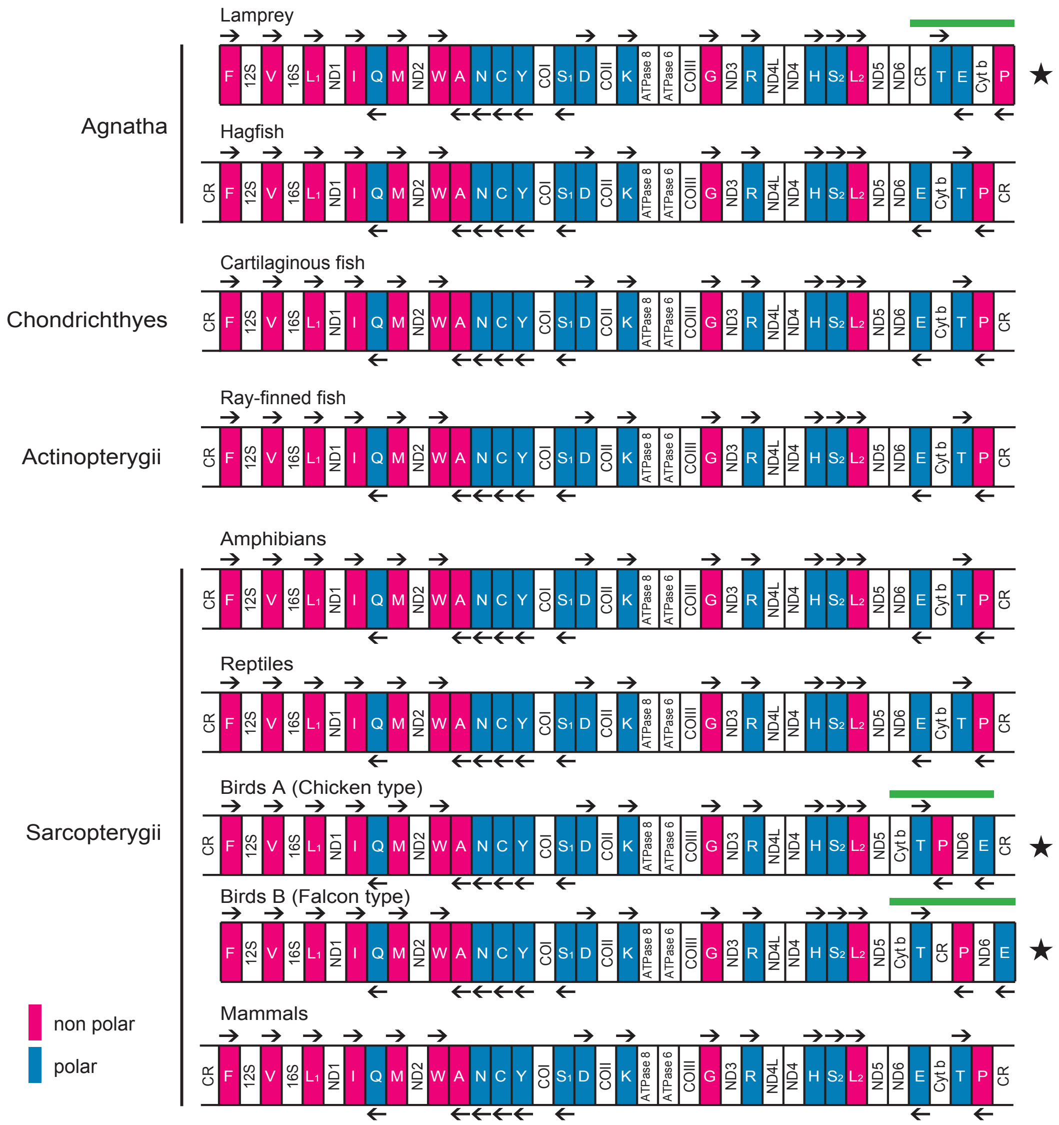

Supplementary Fig. S1

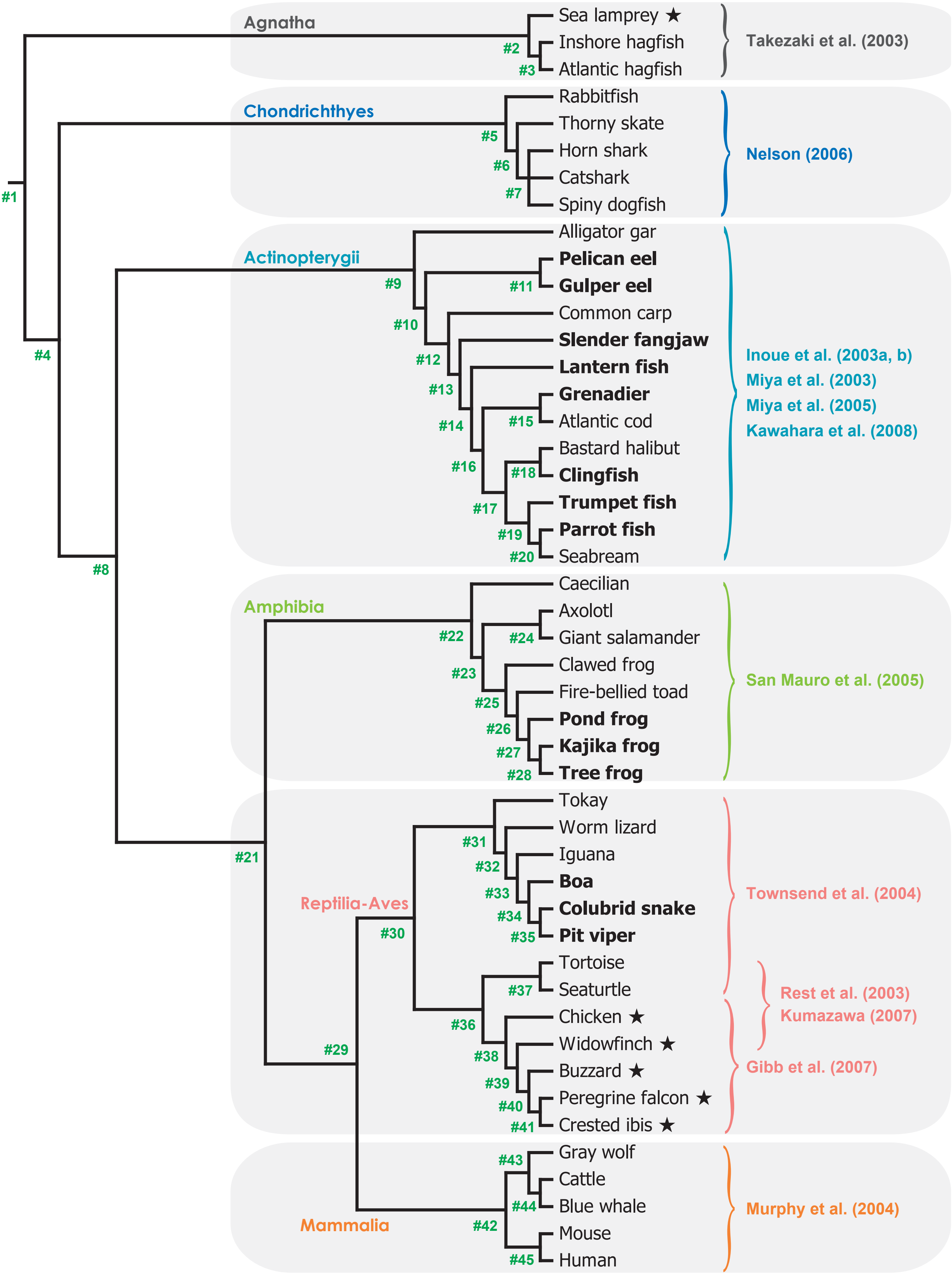

Supplementary Fig. S2

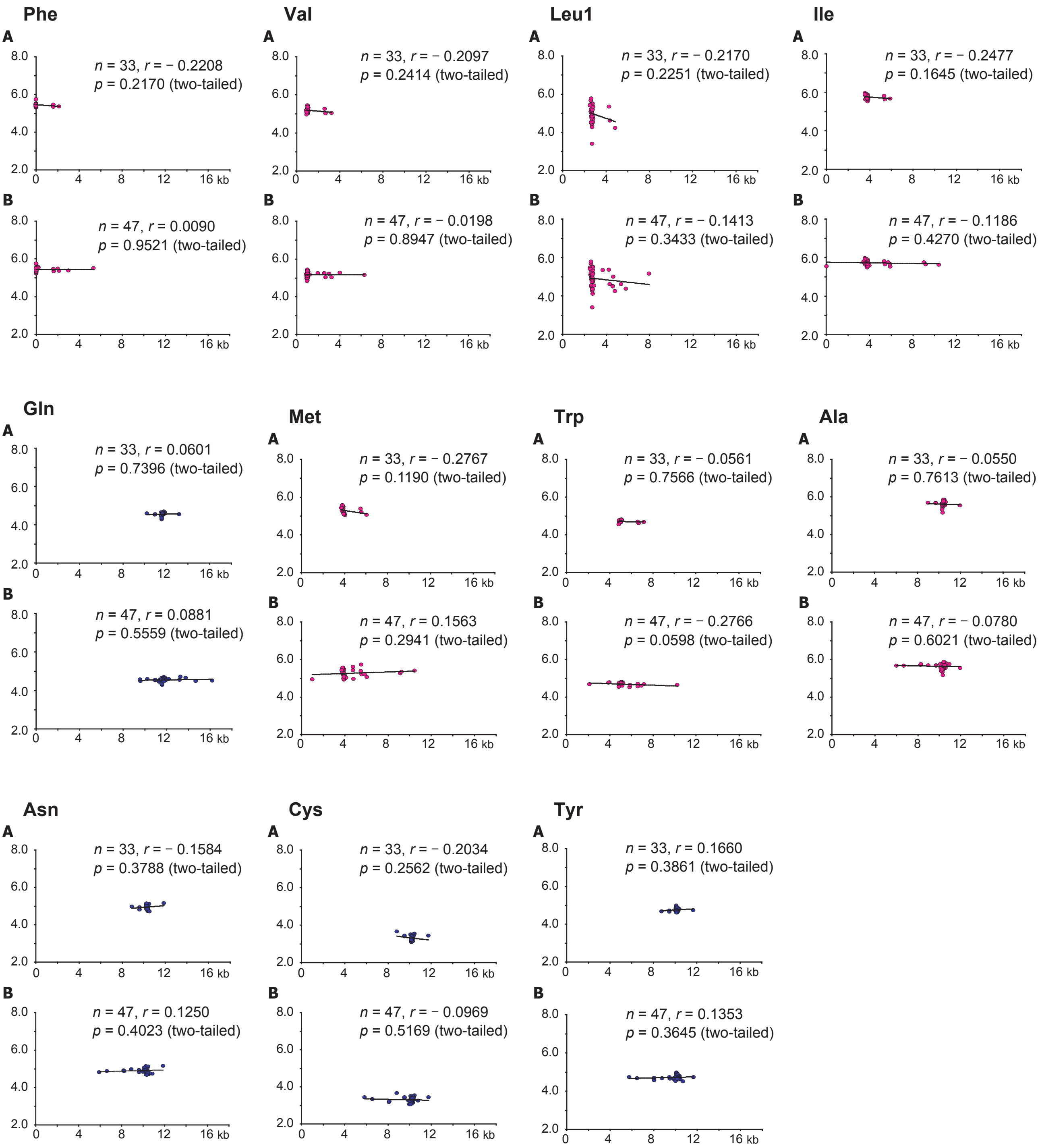

Supplementary Fig. S3

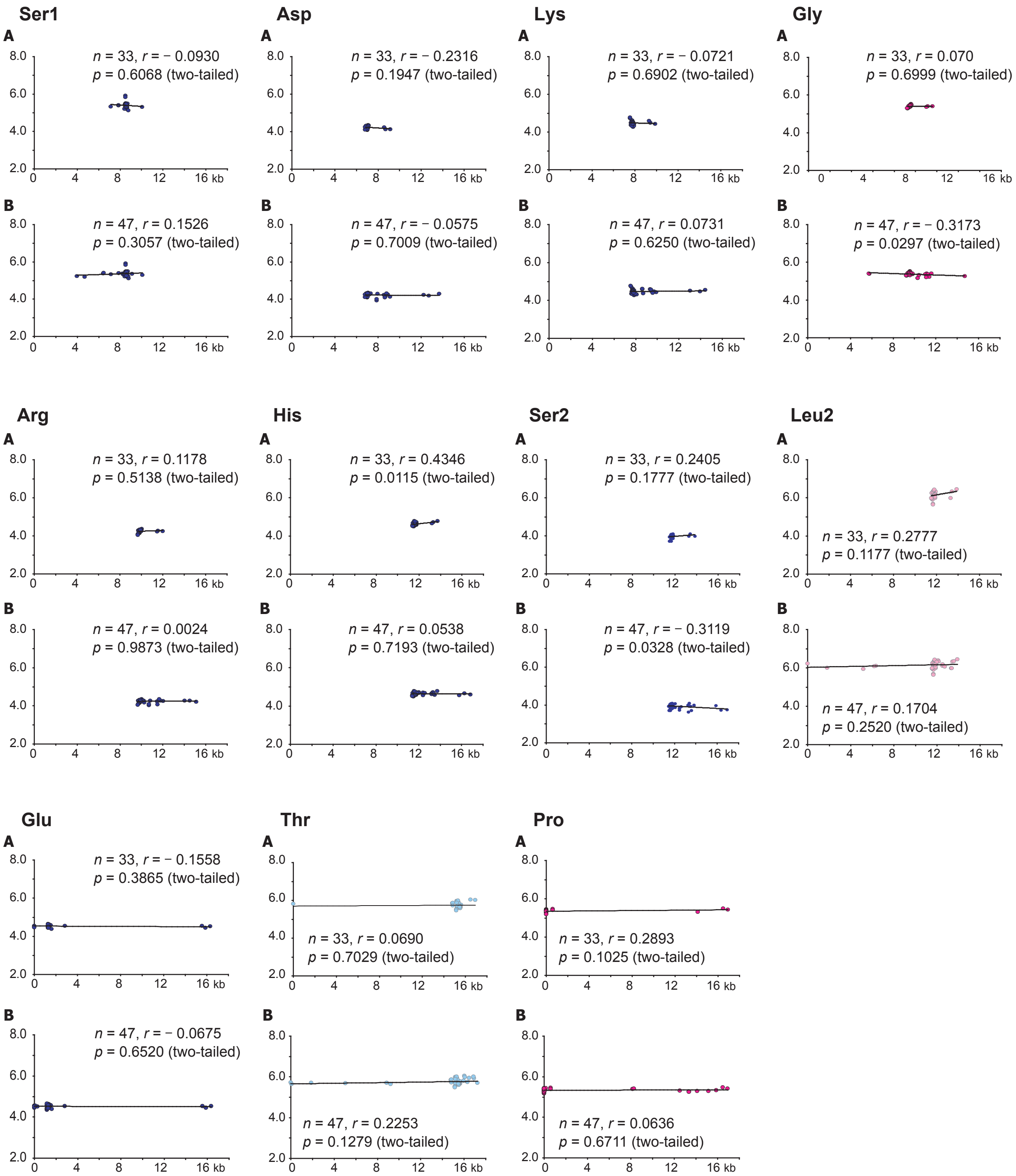

Supplementary Fig. S4

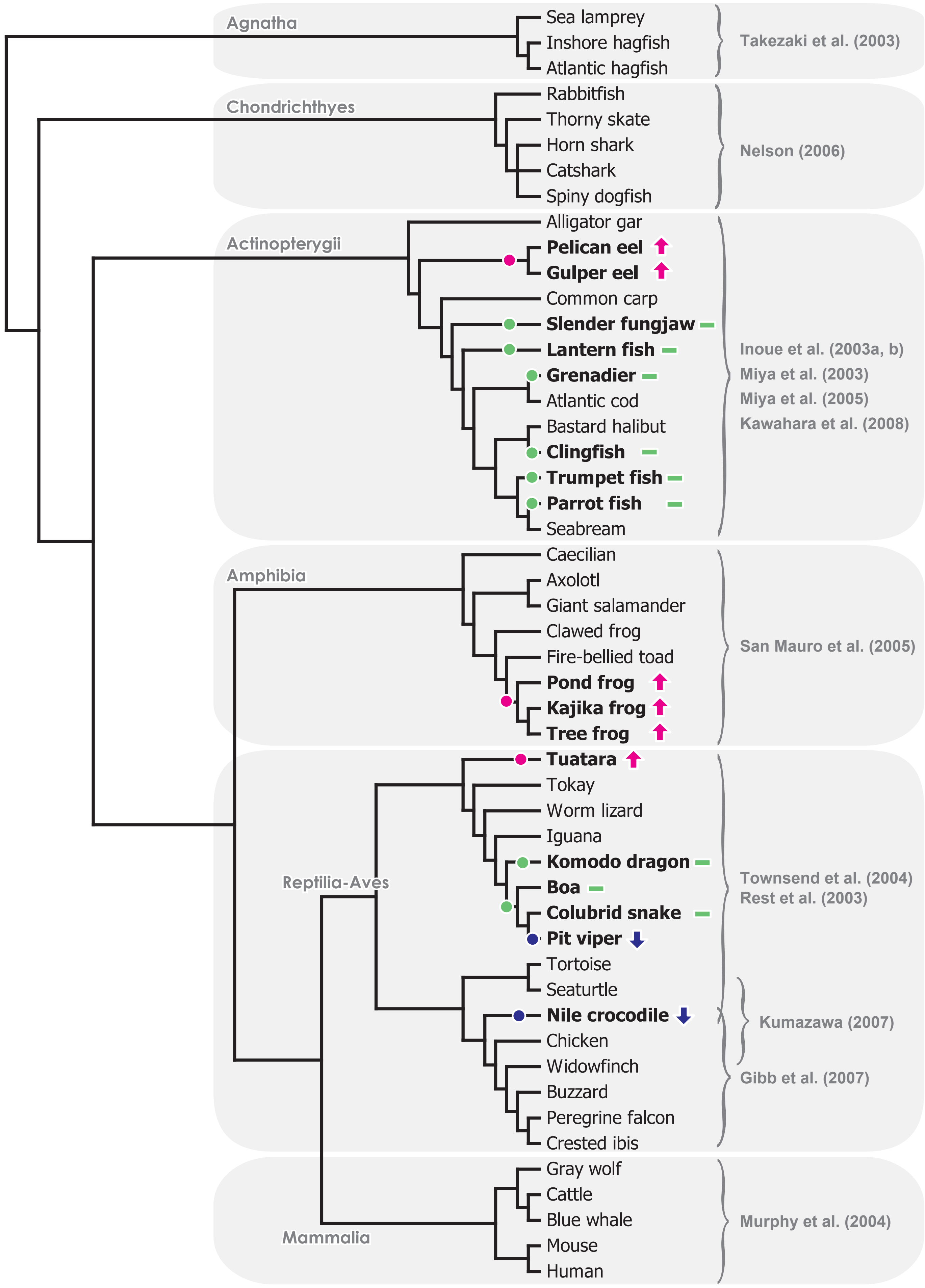

Supplementary Fig. S5

Supplementary references

[a] Inoue JG, Miya M, Tsukamoto K, Nishida M: **Basal actinopterygian relationships: a mitogenomic perspective on the phylogeny of the "ancient fish".** *Mol Phylogenet Evol* 2003, **26**:110-120

[b] Inoue JG, Miya M, Tsukamoto K, Nishida M: **Evolution of the deep-sea gulper eel mitochondrial genomes: large-scale gene rearrangements originated within the eels.** *Mol Biol Evol* 2003, **20**:1917-1924.

Kawahara R, Miya M, Mabuchi K, Lavoué S, Inoue JG, Satoh TP, Kawaguchi A, Nishida M: **Interrelationships of the 11 gasterosteiform families (sticklebacks, pipefishes, and their relatives): a new perspective based on whole mitogenome sequences from 75 higher teleosts.** *Mol Phylogenet Evol* 2008, **46**:224-236.

Miya M, Takeshima H, Endo H, Ishiguro NB, Inoue JG, Mukai T, Satoh TP, Yamaguchi M, Kawaguchi A, Mabuchi K, Shirai SM, Nishida M: **Major patterns of higher teleostean phylogenies: a new perspective based on 100 complete mitochondrial DNA sequences.** *Mol Phylogenet Evol* 2003, **26**:121-138.

Miya M, Satoh TP, Nishida M: **The phylogenetic position of toadfishes (order Batrachoidiformes) in the higher ray-finned fish as inferred from partitioned Bayesian analysis of 102 whole mitochondrial genome sequences.** *Biol J Linn Soc Lond* 2005, **85**:289-306.

Rest JS, Ast JC, Austin CC, Waddell PJ, Tibbetts EA, Hay JM, Mindell DP: **Molecular systematics of primary reptilian lineages and the tuatara mitochondrial genome.** *Mol Phylogenet Evol* 2003, **29**:289-297.

Takezaki N, Figueroa F, Zaleska-Rutczynska Z, Klein J: **Molecular phylogeny of early vertebrates: monophyly of the agnathans as revealed by sequences of 35 genes.** *Mol Biol Evol* 2003, **20**:287-292.

Murphy WJ, Pevzner PA, O'Brien SJ: **Mammalian phylogenomics comes of age.** *Trends Genet* 2004, **20**:631-639.

Townsend TM, Larson A, Louis E, Macey JR: **Molecular phylogenetics of squamata: the position of snakes, amphisbaenians, and dibamids, and the root of the squamate tree.** *Syst Biol* 2004, **53**:735-757.

San Mauro D, Vences M, Alcobendas M, Zardoya R, Meyer A: **Initial diversification of living amphibians predated the breakup of Pangea.** *Am Nat* 2005, **165**:590-599.

Nelson JS: *Fishes of the World, 4th Edition*. New Jersey: John Wiley & Sons Inc; 2006.

Gibb GC, Kardailsky O, Kimball RT, Braun EL, Penny D: **Mitochondrial genomes and avian phylogeny: complex characters and resolvability without explosive radiations.** *Mol Biol Evol* 2007, **24**:269-280.

Kumazawa Y: **Mitochondrial genomes from major lizard families suggest their phylogenetic relationships and ancient radiations.** *Gene* 2007, **388**:19-26.

Supplementaly tables

Table S1 - List of the inferred ancestral states of tRNA position (base pair distance from the 3´-end of control region) and usage of codons corresponding to each tRNA (logarithm of the absolute number), from the analysis based on independent contrasts using 33 evolutionarily stable vertebrate mitochondrial genomes

tRNA	Node name [a]	tRNA position	Codon usage
Leu (CUN)	#1	12836	6.0753
	#3	11533	5.9670
	#5	11849	6.1410
	#7	11868	6.0831
	#12	11861	6.2097
	#17	11902	6.3300
	#21	11653	6.0337
	#24	11667	5.6610
	#25	11678	6.0065
	#30	11736	6.2734
	#32	11632	6.2740
	#37	11766	6.1360
	#39	12452	6.4303
	#41	12582	6.3575
	#44	11696	6.1950
	#45	11678	6.2075
Phe	#1	1	5.4557
	#3	1	5.7380
	#5	1	5.4667
	#7	1	5.4856
	#12	1	5.4277
	#17	1	5.4785
	#21	1	5.4427
	#24	1	5.5230
	#25	1	5.4465
	#30	1	5.3668
	#32	1	5.3460
	#37	1	5.3630
	#39	13	5.3907
	#41	40	5.3495
	#44	1	5.4745
	#45	1	5.4320
Val	#1	1904	5.2723
	#3	929	5.0255
	#5	1026	5.2397

	#7	1024	5.2124
	#12	1021	5.4293
	#17	1020	5.3540
	#21	1011	5.1360
	#24	992	5.1505
	#25	974	5.2550
	#30	1041	5.0678
	#32	1049	5.1090
	#37	1042	5.0270
	#39	1528	5.2503
	#41	1677	5.0710
	#44	1035	5.2495
	#45	1025	5.1240
Leu (UUR)	#3	2544	5.4270
	#5	2767	5.3453
	#7	2770	5.4079
	#8	2725	4.9664
	#12	2769	4.8780
	#17	2796	4.6215
	#24	2627	5.7225
	#25	2627	5.3020
	#18	2682	5.0350
	#32	2619	4.8120
	#36	2731	4.6713
	#39	3294	3.6787
	#41	3443	4.4340
	#44	2674	4.7900
	#45	2664	4.7295
Ile	#1	4792	5.7797
	#3	3576	5.9275
	#5	3817	5.7920
	#7	3822	5.8267
	#12	3821	5.6337
	#17	3851	5.6395
	#21	3676	5.8020
	#24	3672	5.8345
	#25	3670	5.7765
	#30	3763	5.7110
	#32	3665	5.6690
	#37	3773	5.7715
	#39	4388	5.6793
	#41	4537	5.6400
	#44	3707	5.7745
	#45	3696	5.8450

Met	#1	4934	5.2990
	#3	3720	5.4250
	#5	3959	5.2080
	#7	3994	5.1294
	#12	3964	5.1723
	#17	4024	5.0590
	#21	3816	5.5483
	#24	3814	5.5110
	#25	3809	5.2435
	#30	3909	5.2800
	#32	3803	5.3405
	#37	3911	5.4785
	#39	4540	5.0897
	#41	4689	5.1695
	#44	3849	5.4745
	#45	3835	5.4210
Trp	#1	6059	4.7287
	#3	4842	4.5485
	#5	5071	4.7790
	#7	5080	4.7896
	#12	5078	4.7870
	#17	5103	4.7875
	#14	4911	4.7039
	#25	4920	4.7490
	#18	4977	4.6440
	#32	4910	4.6100
	#36	5034	4.6897
	#39	5668	4.6663
	#41	5814	4.6390
	#44	4959	4.6490
	#45	4942	4.6395
Ala	#3	10322	5.2465
	#5	11438	5.5827
	#7	10434	5.6424
	#8	10434	5.6834
	#12	10457	5.8277
	#17	10441	5.8505
	#24	10520	5.5620
	#25	10389	5.6650
	#18	10319	5.5811
	#32	10493	5.6860
	#36	10348	5.5634
	#39	10171	5.7430

	#41	10037	5.6680
	#45	10353	5.5040
Gly	#1	10534	5.3927
	#3	9246	5.2880
	#5	9557	5.3973
	#7	9570	5.4311
	#12	9566	5.5117
	#17	9595	5.4830
	#21	9377	5.3723
	#24	9382	5.3980
	#25	9386	5.4205
	#30	9452	5.3566
	#32	9349	5.3460
	#37	9466	5.3840
	#39	10151	5.4120
	#41	10280	5.4050
	#44	9414	5.3705
	#45	9405	5.3570
Pro	#1	1	5.3383
	#2	309	5.2462
	#3	1	5.1760
	#5	1	5.3260
	#7	1	5.3537
	#12	1	5.3647
	#17	1	5.3935
	#21	1	5.2557
	#24	1	5.2705
	#25	1	5.3280
	#30	1	5.3508
	#32	1	5.3820
	#36	15	5.4100
	#37	1	5.3725
	#39	1861	5.4030
	#41	3154	5.4350
	#44	1	5.3005
	#45	1	5.3335
Gln	#3	11580	4.3170
	#5	12695	4.5710
	#7	11689	4.5979
	#8	11682	4.6668
	#12	11712	4.5910
	#17	11661	4.5535
	#24	11766	4.5790
	#25	11638	4.6000

	#30	11569	4.5075
	#32	11737	4.6445
	#39	11426	4.5537
	#41	11299	4.5590
	#43	11610	4.4932
	#45	11603	4.4470
Asn	#3	10260	5.0600
	#5	11365	5.0967
	#7	10362	4.9573
	#8	10357	4.8832
	#12	10384	4.7670
	#17	10368	4.7535
	#24	10446	5.0230
	#25	10317	4.9470
	#18	10245	4.9915
	#32	10415	4.8295
	#36	10276	4.9469
	#39	10100	4.8360
	#41	9962	4.9050
	#44	10275	5.0625
	#45	10275	5.1120
Cys	#1	9195	3.5589
	#3	10188	3.2575
	#5	11256	3.3343
	#7	10254	3.1774
	#9	10304	3.3623
	#11	10309	3.2956
	#24	10347	3.4655
	#25	10219	3.4175
	#18	10150	3.3300
	#32	10332	3.3520
	#36	10193	3.3404
	#39	10027	3.3057
	#41	9887	3.3265
	#45	10183	3.2115
Tyr	#1	9234	4.7057
	#3	10117	4.6755
	#5	11193	4.7530
	#7	10183	4.7860
	#12	10212	4.7240
	#17	10224	4.6675
	#21	10070	4.7100
	#24	10275	4.8395
	#25	10148	4.7175

	#32	10260	4.7435
	#36	10126	4.7641
	#39	9957	4.6847
	#41	9818	4.7225
	#43	10113	4.9346
	#45	10117	4.8665
Ser (UCN)	#1	7499	5.3567
	#3	8502	5.8670
	#5	9566	5.3030
	#7	8556	5.3700
	#9	8610	5.1947
	#11	8621	5.2850
	#24	8656	5.4550
	#25	8523	5.4360
	#30	8468	5.3142
	#32	8639	5.2100
	#37	8506	5.4680
	#39	8342	5.4347
	#41	8201	5.4265
	#43	8498	5.4136
	#45	8497	5.4395
Asp	#3	6802	4.1795
	#5	7092	4.2387
	#7	7098	4.2560
	#8	7084	4.2907
	#12	7087	4.3397
	#17	7126	4.3170
	#14	6906	4.1717
	#25	6933	4.2760
	#32	6905	4.1665
	#36	7025	4.1221
	#39	7667	4.1983
	#41	7817	4.1270
	#28	6951	4.2414
	#44	6960	4.2270
Lys	#3	7551	4.7490
	#5	7853	4.4957
	#7	7867	4.4374
	#8	7850	4.4636
	#12	7861	4.3170
	#17	7895	4.3305
	#14	7657	4.4754
	#25	7689	4.3870
	#30	7762	4.5258

	#32	7662	4.3940
	#37	7774	4.4940
	#39	8437	4.4423
	#41	8578	4.4775
	#44	7719	4.5900
	#45	7708	4.5945
Arg	#1	10960	4.2523
	#3	9687	4.0605
	#5	9973	4.2480
	#7	9988	4.2921
	#12	9987	4.3263
	#17	10017	4.3440
	#14	9771	4.2629
	#25	9799	4.2535
	#18	9843	4.2040
	#32	9760	4.2625
	#36	9891	4.2647
	#39	10569	4.2853
	#41	10705	4.2765
	#44	9828	4.1740
	#45	9818	4.1510
His	#1	12700	4.6310
	#3	11407	4.5380
	#5	11708	4.6250
	#7	11726	4.6371
	#12	11731	4.6537
	#17	11755	4.7000
	#14	11509	4.4997
	#25	11539	4.5845
	#18	11574	4.6625
	#32	11493	4.6820
	#36	11631	4.6873
	#39	12320	4.6737
	#41	12450	4.7360
	#44	11562	4.5590
	#45	11551	4.5750
Ser (AGY)	#3	11470	3.8230
	#5	11786	3.9900
	#7	11797	4.0196
	#8	11722	3.8978
	#12	11790	3.9987
	#17	11825	4.0070
	#24	11609	3.8605
	#25	11609	4.0605

	#18	11643	3.9410
	#32	11562	3.8900
	#36	11701	3.9739
	#39	12390	4.0250
	#41	12519	4.0860
	#44	11632	4.0515
	#45	11620	3.9310
Glu	#3	1321	4.4715
	#5	2212	4.5603
	#7	1293	4.6007
	#8	1304	4.6110
	#12	1319	4.6017
	#17	1289	4.5950
	#24	1466	4.5485
	#25	1309	4.5540
	#30	73	4.4758
	#32	1436	4.4355
	#37	1292	4.4770
	#39	25	4.4767
	#41	126	4.4715
	#43	1282	4.5432
	#45	1283	4.4940
Thr	#1	25	5.7420
	#3	15161	5.5030
	#5	15486	5.6730
	#7	15508	5.6340
	#12	15501	5.7013
	#17	15545	5.6525
	#21	15230	5.8263
	#24	15253	5.6255
	#25	15283	5.7380
	#30	15096	5.9246
	#32	15245	5.9940
	#37	15375	5.8900
	#39	15506	5.7960
	#41	15631	5.9050
	#44	15314	5.7710
	#45	15298	5.7880

[a] Positions of the nodes in a composite tree of the sampled species were shown in supplementary Fig. S2.

Table S2 - List of the inferred ancestral states of tRNA position (base pair distance from the 3´ -end of control region) and usage of codons corresponding to each tRNA (logarithm of the absolute number), from the analysis based on independent contrasts using 14 species with rearranged mitochondrial gene order

tRNA	Node name [a]	tRNA position	Codon usage
Leu (CUN)	#8	2054	6.1846
	#10	8599	6.2067
	#11	6251	6.0865
	#13	12129	6.3364
	#14	12136	6.2593
	#16	12156	6.1660
	#17	12723	6.1676
	#19	12746	6.3170
	#21	464	6.1617
	#27	25	6.1242
	#28	3061	5.9760
	#30	12547	6.2038
	#34	13012	6.0494
	#35	12525	6.1400
Phe	#8	7	5.3936
	#10	1	5.3637
	#11	1	5.3515
	#13	1	5.3768
	#14	2	5.4397
	#16	6	5.4925
	#17	127	5.4724
	#19	55	5.3935
	#21	43	5.4247
	#27	640	5.5380
	#28	3286	5.4930
	#30	2	5.2972
	#34	1	5.2736
	#35	1	5.2445
Val	#8	1252	5.1395
	#10	1031	5.2226
	#11	1011	5.2700
	#13	1052	5.1714
	#14	1125	5.2773
	#16	1312	5.2438
	#17	1984	5.2818
	#19	2028	5.3190
	#21	1534	5.0531
	#27	2029	5.1792

	#28	4370	5.1840
	#30	1119	4.9113
	#34	991	4.9426
	#35	985	4.8400
Leu (UUR)	#8	3280	4.6213
	#10	2782	4.7288
	#11	2760	5.0875
	#13	2806	4.3418
	#14	2899	4.4257
	#16	3114	4.9341
	#17	3833	4.9454
	#19	4030	4.3565
	#21	3891	4.5097
	#27	3884	4.7410
	#28	6097	5.0695
	#30	3900	4.2494
	#34	5066	4.5666
	#35	4626	4.4940
Ile	#8	5071	5.6505
	#10	5835	5.5886
	#11	9755	5.6400
	#13	3353	5.5333
	#14	2745	5.5984
	#16	1507	5.6232
	#17	345	5.5434
	#19	4762	5.5350
	#21	4383	5.7147
	#27	4983	5.7222
	#28	7162	5.7495
	#30	3793	5.7063
	#34	3529	5.7298
	#35	3486	5.7745
Met	#8	5596	5.1984
	#10	6334	5.1770
	#11	9838	5.3770
	#13	3940	4.9612
	#14	3952	5.0278
	#16	3699	5.0339
	#17	3364	4.9586
	#19	1977	4.9520
	#21	4920	5.2206
	#27	5131	5.0884
	#28	7310	5.2360
	#30	4693	5.3693

	#34	5212	5.6370
	#35	4770	5.5170
Trp	#8	5170	4.6707
	#10	4442	4.7441
	#11	3926	4.7625
	#13	5075	4.7242
	#14	5107	4.7197
	#16	4909	4.6932
	#17	4684	4.6664
	#19	3308	4.7000
	#21	6053	4.5945
	#27	6279	4.6090
	#28	8438	4.6150
	#30	5807	4.5781
	#34	6309	4.5412
	#35	5861	4.5220
Ala	#8	8922	5.6782
	#10	8200	5.7043
	#11	6345	5.6660
	#13	10813	5.7455
	#14	10596	5.7545
	#16	10464	5.7165
	#17	10392	5.7748
	#19	10451	5.8315
	#21	9739	5.6510
	#27	9324	5.7370
	#28	8287	5.7280
	#30	10227	5.5543
	#34	10229	5.4692
	#35	10168	5.4650
Gly	#8	8835	5.3166
	#10	7444	5.3801
	#11	5753	5.3915
	#13	9830	5.3678
	#14	9832	5.3852
	#16	9854	5.3563
	#17	10415	5.3408
	#19	10431	5.3825
	#21	10556	5.2506
	#27	10814	5.2816
	#28	12939	5.2495
	#30	10273	5.2157
	#34	10771	5.1828
	#35	10306	5.1675

Pro	#8	972	5.3582
	#10	708	5.3999
	#11	8209	5.4025
	#13	50	5.3972
	#14	7	5.3457
	#16	143	5.3355
	#17	331	5.3554
	#19	1	5.3890
	#21	1351	5.3149
	#27	14415	5.2838
	#28	13353	5.2625
	#30	94	5.3499
	#34	8	5.2686
	#35	158	5.2695
Gln	#8	11922	4.5616
	#10	12889	4.6214
	#11	13521	4.6770
	#13	12240	4.5613
	#14	12288	4.5069
	#16	12886	4.4825
	#17	14880	4.5088
	#19	13115	4.5055
	#21	10995	4.4995
	#27	10589	4.4806
	#28	9571	4.5205
	#30	11470	4.5207
	#34	11453	4.5454
	#35	11384	4.5325
Asn	#8	8844	4.8294
	#10	8122	4.7906
	#11	6267	4.8360
	#13	10744	4.7416
	#14	10522	4.7507
	#16	10390	4.7325
	#17	10318	4.6982
	#19	10373	4.7090
	#21	9663	4.8697
	#27	9246	4.8450
	#28	8213	4.8825
	#30	10154	4.8975
	#34	10156	4.9500
	#35	10092	4.9950
Cys	#8	8751	3.2759
	#10	8024	3.3354

	#11	6192	3.3830
	#13	10613	3.2840
	#14	10339	3.3260
	#16	10284	3.3744
	#17	10207	3.3318
	#19	10265	3.3315
	#21	9576	3.2142
	#27	9156	3.2342
	#28	8123	3.1985
	#30	10071	3.1916
	#34	10063	3.0876
	#35	9997	3.1515
Tyr	#8	8686	4.6201
	#10	7961	4.6289
	#11	6118	4.6995
	#13	10577	4.5528
	#14	10363	4.6205
	#16	10214	4.6455
	#17	10142	4.6942
	#19	10193	4.6585
	#21	9508	4.6109
	#27	9087	4.6324
	#28	8055	4.6285
	#30	10006	4.5867
	#34	9997	4.5666
	#35	9937	4.6335
Ser (UCN)	#8	6931	5.3164
	#10	6129	5.2578
	#11	4320	5.2090
	#13	8939	5.3104
	#14	8685	5.2513
	#16	8592	5.3099
	#17	8505	5.3050
	#19	8574	5.2495
	#21	7876	5.3773
	#27	7464	5.3824
	#28	6441	5.3935
	#30	8367	5.3715
	#34	8337	5.3310
	#35	8279	5.3280
Asp	#8	8931	4.2216
	#10	9829	4.2622
	#11	13181	4.2400
	#13	7162	4.2862

	#14	7345	4.3011
	#16	7389	4.2965
	#17	7949	4.2844
	#19	7947	4.2760
	#21	8086	4.1794
	#27	8327	4.2760
	#28	10462	4.2340
	#30	7823	4.0707
	#34	8338	4.0442
	#35	7883	3.9695
Lys	#8	9762	4.4645
	#10	10730	4.4439
	#11	14193	4.5100
	#13	7935	4.3725
	#14	8118	4.3378
	#16	8156	4.3924
	#17	8715	4.3690
	#19	8725	4.3690
	#21	8848	4.4860
	#27	9096	4.4446
	#28	11232	4.4830
	#30	8578	4.5325
	#34	9087	4.5830
	#35	8630	4.5950
Arg	#8	11571	4.2158
	#10	12177	4.2416
	#11	14285	4.2700
	#13	10250	4.2110
	#14	10252	4.2709
	#16	10272	4.2611
	#17	10831	4.2820
	#19	10851	4.3105
	#21	10973	4.1890
	#27	11233	4.2368
	#28	13360	4.2410
	#30	10687	4.1352
	#34	11179	4.0598
	#35	10716	4.0595
His	#8	13313	4.6367
	#10	13936	4.6488
	#11	16027	4.6090
	#13	11985	4.6916
	#14	11993	4.6619
	#16	12013	4.6440

	#17	12579	4.6744
	#19	12594	4.6630
	#21	12697	4.6242
	#27	12955	4.5910
	#28	15078	4.5850
	#30	12413	4.6616
	#34	12882	4.6200
	#35	12401	4.6275
Ser (AGY)	#8	13382	3.8548
	#10	14003	3.8956
	#11	16091	3.8540
	#13	12054	3.9405
	#14	12056	4.0530
	#16	12082	4.0125
	#17	12645	3.9078
	#19	12664	3.9015
	#21	12766	3.8124
	#27	13025	3.7792
	#28	15146	3.7375
	#30	12481	3.8498
	#34	12949	3.6962
	#35	12469	3.7835
Glu	#8	384	4.5033
	#10	419	4.5614
	#11	1167	4.6095
	#13	139	4.5096
	#14	47	4.5250
	#16	16	4.4782
	#17	1252	4.4432
	#19	1294	4.4975
	#21	351	4.4429
	#27	1152	4.4018
	#28	1162	4.3940
	#30	92	4.4891
	#34	1233	4.4998
	#35	1212	4.5160
Thr	#8	5001	5.7543
	#10	11823	5.7215
	#11	9054	5.6780
	#13	15765	5.7683
	#14	15427	5.6993
	#16	15801	5.6917
	#17	16363	5.7264
	#19	16408	5.6865

#21	2046	5.7884
#27	332	5.6682
#28	3093	5.6970
#30	15832	5.9236
#34	16558	6.0078
#35	16059	6.0180

[a] Positions of the nodes in a composite tree of the sampled species were shown in supplementary Fig. S2.

Table S3 - List of the inferred ancestral states of tRNA position (base pair distance from the 3´ -end of control region) and usage of codons corresponding to each tRNA (logarithm of the absolute number), from the analysis based on independent contrasts using the all (47 species) vertebrate mitochondrial genomes

tRNA	Node name [a]	tRNA position	Codon usage
Leu (CUN)	#1	11962	6.0530
	#2	12582	5.9802
	#3	11533	5.9670
	#4	11277	6.1380
	#5	11850	6.1310
	#6	11871	6.1231
	#7	11868	6.0831
	#8	10646	6.1461
	#9	10560	6.2229
	#10	8544	6.1782
	#11	6251	6.0865
	#12	11970	6.2771
	#13	12130	6.3439
	#14	12139	6.2779
	#15	11825	6.1610
	#16	12166	6.2070
	#17	12582	6.2613
	#18	12302	6.1980
	#19	12887	6.3288
	#20	11938	6.3135
	#21	10746	6.0579
	#22	9952	5.9310
	#23	7548	5.8154
	#24	11667	5.6610
	#25	4719	5.9819
	#26	1096	6.1192
	#27	25	6.1242
	#28	3061	5.9760
	#29	11748	6.2054
	#30	11808	6.2355
	#31	11745	6.2147
	#32	11805	6.2272
	#33	12178	6.2098
	#34	13012	6.0494
	#35	12525	6.1400
	#36	11878	6.2583

	#37	11766	6.1360
	#38	12000	6.3902
	#39	12368	6.4179
	#40	13352	6.4112
	#41	12582	6.3575
	#42	11690	6.1765
	#43	11703	6.1434
	#44	11696	6.1950
	#45	11678	6.2075
Phe	#1	11	5.5147
	#2	84	5.5712
	#3	1	5.7380
	#4	1	5.4488
	#5	1	5.4690
	#6	1	5.4705
	#7	1	5.4797
	#8	1	5.4254
	#9	1	5.4209
	#10	1	5.3745
	#11	1	5.3515
	#12	1	5.3993
	#13	1	5.3721
	#14	2	5.4282
	#15	1	5.4780
	#16	5	5.4665
	#17	41	5.4530
	#18	15	5.4985
	#19	121	5.4044
	#20	1	5.4485
	#21	1	5.4305
	#22	1	5.4540
	#23	2	5.4895
	#24	1	5.5230
	#25	3	5.4533
	#26	12	5.4925
	#27	640	5.5380
	#28	3286	5.4930
	#29	1	5.4033
	#30	1	5.3563
	#31	1	5.3394
	#32	1	5.3189
	#33	1	5.3446

	#34	1	5.2736
	#35	1	5.2445
	#36	2	5.3750
	#37	1	5.3630
	#38	2	5.3879
	#39	10	5.3862
	#40	436	5.3594
	#41	40	5.3495
	#42	1	5.4484
	#43	1	5.4658
	#44	1	5.4745
	#45	1	5.4320
Val	#1	1354	5.1940
	#2	1717	5.1614
	#3	929	5.0255
	#4	1026	5.2320
	#5	1025	5.2391
	#6	1032	5.2010
	#7	1024	5.2124
	#8	1028	5.2237
	#9	1028	5.3083
	#10	1021	5.2998
	#11	1011	5.2700
	#12	1032	5.3319
	#13	1050	5.1810
	#14	1120	5.3044
	#15	1016	5.3290
	#16	1320	5.3159
	#17	1797	5.3004
	#18	1411	5.2925
	#19	2326	5.3088
	#20	1022	5.3775
	#21	1028	5.1268
	#22	1009	5.1260
	#23	1019	5.1829
	#24	992	5.1505
	#25	1048	5.2179
	#26	1366	5.2677
	#27	2029	5.1792
	#28	4370	5.1840
	#29	1049	5.1277
	#30	1074	5.0772

	#31	1042	5.0743
	#32	1049	5.0926
	#33	1013	5.0321
	#34	991	4.9426
	#35	985	4.8400
	#36	1111	5.0803
	#37	1042	5.0270
	#38	1189	5.1378
	#39	1463	5.2392
	#40	2489	5.0506
	#41	1677	5.0710
	#42	1026	5.1762
	#43	1028	5.2318
	#44	1035	5.2495
	#45	1025	5.1240
Leu (UUR)	#1	3121	5.2809
	#2	3474	5.3820
	#3	2544	5.4270
	#4	2753	5.1629
	#5	2766	5.3556
	#6	2780	5.3691
	#7	2770	5.4079
	#8	2738	4.9389
	#9	2786	4.8214
	#10	2773	4.8381
	#11	2760	5.0875
	#12	2786	4.5691
	#13	2807	4.3275
	#14	2905	4.3892
	#15	2748	5.0625
	#16	3158	4.8935
	#17	3724	4.6936
	#18	3224	4.9405
	#19	4343	4.4302
	#20	2784	4.5220
	#21	2684	5.0736
	#22	2650	5.3107
	#23	2693	5.5110
	#24	2627	5.7225
	#25	2767	5.2830
	#26	3081	4.9841
	#27	3884	4.7410

	#28	6097	5.0695
	#29	2725	4.7982
	#30	2786	4.7847
	#31	2761	5.0155
	#32	2872	4.8788
	#33	3402	4.5521
	#34	5066	4.5666
	#35	4626	4.4940
	#36	2815	4.5311
	#37	2724	4.9070
	#38	2915	4.1257
	#39	3220	3.7522
	#40	4222	4.3140
	#41	3443	4.4340
	#42	2668	4.8110
	#43	2672	4.8980
	#44	2674	4.7900
	#45	2664	4.7295
Ile	#1	4271	5.8152
	#2	4551	5.8576
	#3	3576	5.9275
	#4	3965	5.7657
	#5	3817	5.7948
	#6	3830	5.8263
	#7	3822	5.8267
	#8	4144	5.7319
	#9	4560	5.6866
	#10	6158	5.6326
	#11	9755	5.6400
	#12	3748	5.6246
	#13	3624	5.5283
	#14	3361	5.5863
	#15	3797	5.6030
	#16	2474	5.5923
	#17	1490	5.5796
	#18	481	5.6075
	#19	4979	5.5498
	#20	3835	5.5750
	#21	3714	5.7838
	#22	3692	5.7938
	#23	3745	5.8113
	#24	3672	5.8345

	#25	3826	5.7863
	#26	4151	5.7270
	#27	4983	5.7222
	#28	7162	5.7495
	#29	3739	5.7722
	#30	3781	5.7128
	#31	3698	5.6904
	#32	3630	5.6634
	#33	3635	5.7158
	#34	3529	5.7298
	#35	3486	5.7745
	#36	3874	5.7374
	#37	3773	5.7715
	#38	3986	5.7006
	#39	4312	5.6740
	#40	5309	5.6580
	#41	4537	5.6400
	#42	3700	5.8293
	#43	3705	5.8126
	#44	3707	5.7745
	#45	3696	5.8450
Met	#1	4418	5.3295
	#2	4697	5.3950
	#3	3720	5.4250
	#4	4114	5.2531
	#5	3963	5.2051
	#6	3986	5.1051
	#7	3994	5.1294
	#8	4297	5.3089
	#9	4714	5.1799
	#10	6337	5.2384
	#11	9838	5.3770
	#12	3942	5.0889
	#13	3895	4.9655
	#14	3833	5.0403
	#15	3936	5.1340
	#16	3367	5.0699
	#17	2800	4.9940
	#18	4376	5.0255
	#19	1739	4.9604
	#20	3978	5.0000
	#21	3864	5.4569

	#22	3831	5.4995
	#23	3886	5.3777
	#24	3814	5.5110
	#25	3965	5.2339
	#26	4295	5.1626
	#27	5131	5.0884
	#28	7310	5.2360
	#29	3901	5.4075
	#30	3964	5.3561
	#31	3917	5.4033
	#32	3969	5.4344
	#33	4318	5.3736
	#34	5212	5.6370
	#35	4770	5.5170
	#36	4017	5.3042
	#37	3911	5.4785
	#38	4135	5.1162
	#39	4463	5.1035
	#40	5464	5.1092
	#41	4689	5.1695
	#42	3841	5.4569
	#43	3848	5.4952
	#44	3849	5.4745
	#45	3835	5.4210
Trp	#1	5429	4.6927
	#2	5831	4.6340
	#3	4842	4.5485
	#4	4995	4.7612
	#5	5071	4.7807
	#6	5089	4.7834
	#7	5080	4.7896
	#8	4909	4.7386
	#9	4854	4.7895
	#10	4438	4.7638
	#11	3926	4.7625
	#12	5065	4.7652
	#13	5034	4.7299
	#14	5000	4.7346
	#15	5059	4.7485
	#16	4600	4.7321
	#17	4111	4.7127
	#18	5494	4.7200

	#19	3017	4.7050
	#20	5131	4.7530
	#21	4972	4.6803
	#22	4939	4.7083
	#23	5001	4.7231
	#24	4925	4.7135
	#25	5084	4.7334
	#26	5430	4.6872
	#27	6279	4.6090
	#28	8438	4.6150
	#29	5011	4.6477
	#30	5075	4.6529
	#31	5026	4.6235
	#32	5079	4.5903
	#33	5434	4.5989
	#34	6309	4.5412
	#35	5861	4.5220
	#36	5130	4.6853
	#37	5019	4.6955
	#38	5253	4.6742
	#39	5589	4.6616
	#40	6587	4.6594
	#41	5814	4.6390
	#42	4950	4.6427
	#43	4959	4.6460
	#44	4959	4.6490
	#45	4942	4.6395
Ala	#1	10016	5.5657
	#2	9490	5.5150
	#3	10322	5.2465
	#4	10668	5.6248
	#5	11350	5.5875
	#6	10466	5.6422
	#7	10434	5.6424
	#8	9926	5.6682
	#9	9574	5.7512
	#10	8112	5.7266
	#11	6345	5.6660
	#12	10573	5.7919
	#13	10817	5.7561
	#14	10604	5.7827
	#15	10514	5.7675

	#16	10471	5.7912
	#17	10420	5.8192
	#18	10394	5.7945
	#19	10448	5.8456
	#20	10451	5.8270
	#21	10345	5.5730
	#22	10343	5.5781
	#23	10380	5.6084
	#24	10520	5.5620
	#25	10231	5.6584
	#26	9957	5.7204
	#27	9324	5.7370
	#28	8287	5.7280
	#29	10348	5.5671
	#30	10344	5.6217
	#31	10367	5.6545
	#32	10508	5.6698
	#33	10305	5.5774
	#34	10229	5.4692
	#35	10168	5.4650
	#36	10319	5.5857
	#37	10353	5.4865
	#38	10283	5.6927
	#39	10188	5.7358
	#40	9834	5.6794
	#41	10037	5.6680
	#42	10353	5.5146
	#43	10353	5.5258
	#44	10353	5.5270
	#45	10353	5.5040
Gly	#1	9826	5.3646
	#2	10283	5.3294
	#3	9246	5.2880
	#4	9319	5.4057
	#5	9557	5.3992
	#6	9574	5.4375
	#7	9570	5.4311
	#8	9050	5.4133
	#9	8733	5.4503
	#10	7386	5.4261
	#11	5753	5.3915
	#12	9672	5.4633

	#13	9831	5.3763
	#14	9835	5.4080
	#15	9533	5.4335
	#16	9865	5.4171
	#17	10272	5.3977
	#18	9997	5.3970
	#19	10574	5.3984
	#20	9624	5.4395
	#21	9427	5.3708
	#22	9393	5.3793
	#23	9473	5.3941
	#24	9382	5.3980
	#25	9573	5.3899
	#26	9930	5.3834
	#27	10814	5.2816
	#28	12939	5.2495
	#29	9467	5.3608
	#30	9521	5.3566
	#31	9469	5.3304
	#32	9527	5.3037
	#33	9902	5.3128
	#34	10771	5.1828
	#35	10306	5.1675
	#36	9578	5.3853
	#37	9466	5.3840
	#38	9701	5.3868
	#39	10068	5.4123
	#40	11048	5.3984
	#41	10280	5.4050
	#42	9415	5.3648
	#43	9426	5.3732
	#44	9414	5.3705
	#45	9405	5.3570
Pro	#1	29	5.2907
	#2	309	5.2462
	#3	1	5.1760
	#4	2	5.3427
	#5	1	5.3283
	#6	1	5.3527
	#7	1	5.3537
	#8	4	5.3595
	#9	7	5.4146

	#10	206	5.3889
	#11	8209	5.4025
	#12	4	5.3741
	#13	34	5.4019
	#14	2	5.3574
	#15	9	5.3515
	#16	10	5.3673
	#17	12	5.3860
	#18	126	5.3745
	#19	1	5.3982
	#20	1	5.3640
	#21	2	5.2963
	#22	1	5.2668
	#23	2	5.2932
	#24	1	5.2705
	#25	4	5.3177
	#26	40	5.3172
	#27	14415	5.2838
	#28	13353	5.2625
	#29	2	5.3305
	#30	5	5.3553
	#31	1	5.3103
	#32	1	5.3627
	#33	2	5.3427
	#34	8	5.2686
	#35	158	5.2695
	#36	26	5.4048
	#37	1	5.3725
	#38	893	5.4396
	#39	1703	5.4065
	#40	8646	5.4266
	#41	3154	5.4350
	#42	1	5.3067
	#43	1	5.2780
	#44	1	5.3005
	#45	1	5.3335
Gln	#1	11416	4.5317
	#2	10743	4.4778
	#3	11580	4.3170
	#4	12255	4.5946
	#5	12608	4.5726
	#6	11721	4.6021

	#7	11689	4.5979
	#8	11857	4.6202
	#9	12095	4.6662
	#10	12702	4.6381
	#11	13521	4.6770
	#12	11874	4.5962
	#13	12189	4.5657
	#14	12155	4.5176
	#15	11772	4.5045
	#16	12570	4.5096
	#17	13583	4.5157
	#18	13760	4.5165
	#19	13397	4.5148
	#20	11667	4.5535
	#21	11591	4.5674
	#22	11582	4.6209
	#23	11628	4.5797
	#24	11766	4.5790
	#25	11482	4.5805
	#26	11216	4.5572
	#27	10589	4.4806
	#28	9571	4.5205
	#29	11601	4.5052
	#30	11595	4.5425
	#31	11614	4.5300
	#32	11746	4.6336
	#33	11545	4.6005
	#34	11453	4.5454
	#35	11384	4.5325
	#36	11573	4.5563
	#37	11603	4.6150
	#38	11542	4.4930
	#39	11444	4.5561
	#40	11088	4.5434
	#41	11299	4.5590
	#42	11606	4.4694
	#43	11610	4.4932
	#44	11603	4.4995
	#45	11603	4.4470
Asn	#1	9945	5.0039
	#2	9422	5.0102
	#3	10260	5.0600

#4	10593	4.9965
#5	11278	5.0835
#6	10393	4.9730
#7	10362	4.9573
#8	9849	4.8953
#9	9498	4.8092
#10	8033	4.8041
#11	6267	4.8360
#12	10502	4.7696
#13	10749	4.7414
#14	10530	4.7504
#15	10441	4.7405
#16	10398	4.7293
#17	10347	4.7160
#18	10322	4.7310
#19	10373	4.7000
#20	10373	4.7405
#21	10268	4.9942
#22	10264	4.9904
#23	10306	4.9910
#24	10446	5.0230
#25	10158	4.9565
#26	9882	4.8684
#27	9246	4.8450
#28	8213	4.8825
#29	10274	4.9987
#30	10271	4.9077
#31	10294	4.8791
#32	10432	4.8015
#33	10226	4.9494
#34	10156	4.9500
#35	10092	4.9950
#36	10247	4.9391
#37	10280	5.0300
#38	10211	4.8410
#39	10116	4.8491
#40	9763	4.8444
#41	9962	4.9050
#42	10277	5.0862
#43	10278	5.0586
#44	10275	5.0625

	#45	10275	5.1120
Cys	#1	9863	3.4272
	#2	9356	3.5014
	#3	10188	3.2575
	#4	10489	3.3407
	#5	11167	3.3252
	#6	10294	3.1525
	#7	10254	3.1774
	#8	9753	3.3587
	#9	9398	3.3911
	#10	7942	3.3149
	#11	6192	3.3830
	#12	10388	3.2414
	#13	10617	3.2777
	#14	10344	3.3100
	#15	10337	3.3485
	#16	10291	3.3353
	#17	10237	3.3196
	#18	10214	3.2950
	#19	10262	3.3458
	#20	10265	3.3140
	#21	10176	3.3214
	#22	10169	3.3451
	#23	10208	3.4325
	#24	10347	3.4655
	#25	10059	3.3969
	#26	9790	3.3369
	#27	9156	3.2342
	#28	8123	3.1985
	#29	10183	3.2939
	#30	10185	3.3929
	#31	10204	3.4522
	#32	10347	3.3797
	#33	10140	3.1432
	#34	10063	3.0876
	#35	9997	3.1515
	#36	10164	3.3277
	#37	10188	3.2950
	#38	10138	3.3630
	#39	10042	3.3015
	#40	9688	3.3712
	#41	9887	3.3265

	#42	10181	3.1988
	#43	10178	3.1852
	#44	10178	3.1345
	#45	10183	3.2115
Tyr	#1	9790	4.7119
	#2	9280	4.6740
	#3	10117	4.6755
	#4	10422	4.7561
	#5	11103	4.7556
	#6	10222	4.7866
	#7	10183	4.7860
	#8	9682	4.7568
	#9	9326	4.7405
	#10	7871	4.6863
	#11	6118	4.6995
	#12	10330	4.6720
	#13	10582	4.5536
	#14	10374	4.6235
	#15	10270	4.6485
	#16	10227	4.6572
	#17	10175	4.6674
	#18	10148	4.6715
	#19	10205	4.6630
	#20	10229	4.6915
	#21	10107	4.7755
	#22	10098	4.7363
	#23	10136	4.7830
	#24	10275	4.8395
	#25	9988	4.7220
	#26	9720	4.6574
	#27	9087	4.6324
	#28	8055	4.6285
	#29	10117	4.8211
	#30	10119	4.7395
	#31	10140	4.7205
	#32	10276	4.7376
	#33	10071	4.6432
	#34	9997	4.5666
	#35	9937	4.6335
	#36	10096	4.7603
	#37	10117	4.8400
	#38	10074	4.6744

	#39	9972	4.6929
	#40	9620	4.6814
	#41	9818	4.7225
	#42	10115	4.8995
	#43	10113	4.9346
	#44	10107	4.8815
	#45	10117	4.8665
Ser (UCN)	#1	8150	5.4350
	#2	7659	5.5466
	#3	8502	5.8670
	#4	8763	5.3048
	#5	9476	5.3129
	#6	8595	5.3365
	#7	8556	5.3700
	#8	8002	5.2954
	#9	7599	5.1994
	#10	6049	5.2303
	#11	4320	5.2090
	#12	8700	5.2533
	#13	8944	5.3073
	#14	8693	5.2421
	#15	8652	5.2955
	#16	8602	5.2913
	#17	8544	5.2862
	#18	8514	5.3125
	#19	8575	5.2582
	#20	8570	5.2825
	#21	8491	5.4055
	#22	8484	5.4294
	#23	8514	5.4533
	#24	8656	5.4550
	#25	8363	5.4514
	#26	8091	5.3779
	#27	7464	5.3824
	#28	6441	5.3935
	#29	8498	5.3777
	#30	8499	5.3265
	#31	8514	5.2019
	#32	8646	5.1903
	#33	8438	5.3169
	#34	8337	5.3310
	#35	8279	5.3280

	#36	8482	5.4634
	#37	8506	5.4680
	#38	8456	5.4584
	#39	8357	5.4365
	#40	8002	5.4070
	#41	8201	5.4265
	#42	8498	5.4270
	#43	8498	5.4136
	#44	8493	5.3635
	#45	8497	5.4395
Asp	#1	7561	4.2239
	#2	7813	4.2038
	#3	6802	4.1795
	#4	7277	4.2473
	#5	7092	4.2407
	#6	7100	4.2513
	#7	7098	4.2560
	#8	7499	4.2549
	#9	7990	4.3112
	#10	9801	4.2760
	#11	13181	4.2400
	#12	7119	4.3149
	#13	7161	4.2889
	#14	7344	4.3084
	#15	7066	4.3305
	#16	7392	4.3163
	#17	7797	4.2996
	#18	7533	4.3035
	#19	8089	4.2954
	#20	7155	4.2825
	#21	6972	4.1903
	#22	6940	4.1839
	#23	7014	4.2850
	#24	6926	4.2835
	#25	7111	4.2867
	#26	7457	4.2588
	#27	8327	4.2760
	#28	10462	4.2340
	#29	7010	4.1978
	#30	7072	4.1545
	#31	7024	4.1753
	#32	7083	4.1514

	#33	7452	4.1149
	#34	8338	4.0442
	#35	7883	3.9695
	#36	7124	4.1318
	#37	7016	4.0945
	#38	7243	4.1719
	#39	7587	4.1929
	#40	8584	4.1270
	#41	7817	4.1270
	#42	6952	4.2394
	#43	6965	4.2312
	#44	6960	4.2270
	#45	6940	4.2470
Lys	#1	8332	4.5727
	#2	8578	4.6566
	#3	7551	4.7490
	#4	8052	4.4748
	#5	7854	4.4906
	#6	7865	4.4407
	#7	7867	4.4374
	#8	8289	4.4564
	#9	8809	4.4168
	#10	10705	4.4341
	#11	14193	4.5100
	#12	7895	4.3522
	#13	7934	4.3656
	#14	8119	4.3188
	#15	7832	4.3350
	#16	8162	4.3455
	#17	8570	4.3580
	#18	8296	4.3430
	#19	8872	4.3740
	#20	7923	4.3440
	#21	7729	4.5019
	#22	7692	4.4656
	#23	7774	4.4452
	#24	7685	4.4715
	#25	7871	4.4169
	#26	8220	4.3747
	#27	9096	4.4446
	#28	11232	4.4830
	#29	7773	4.5442

	#30	7829	4.4895
	#31	7783	4.4913
	#32	7841	4.4077
	#33	8207	4.4895
	#34	9087	4.5830
	#35	8630	4.5950
	#36	7881	4.4876
	#37	7774	4.4940
	#38	7999	4.4807
	#39	8356	4.4495
	#40	9341	4.4424
	#41	8578	4.4775
	#42	7719	4.5967
	#43	7731	4.5990
	#44	7719	4.5900
	#45	7708	4.5945
Arg	#1	10439	4.2051
	#2	10717	4.1562
	#3	9687	4.0605
	#4	10124	4.2622
	#5	9975	4.2550
	#6	9987	4.2662
	#7	9988	4.2921
	#8	10299	4.2704
	#9	10717	4.3031
	#10	12087	4.2782
	#11	14285	4.2700
	#12	10093	4.2871
	#13	10252	4.2159
	#14	10255	4.2849
	#15	9952	4.2825
	#16	10284	4.2991
	#17	10691	4.3187
	#18	10415	4.3100
	#19	10993	4.3280
	#20	10047	4.3040
	#21	9840	4.2330
	#22	9806	4.2566
	#23	9888	4.2453
	#24	9799	4.2625
	#25	9986	4.2268
	#26	10346	4.2862

	#27	11233	4.2368
	#28	13360	4.2410
	#29	9880	4.2056
	#30	9934	4.2509
	#31	9876	4.2356
	#32	9937	4.2382
	#33	10315	4.1756
	#34	11179	4.0598
	#35	10716	4.0595
	#36	9998	4.2677
	#37	9887	4.2555
	#38	10118	4.2808
	#39	10487	4.2869
	#40	11467	4.2594
	#41	10705	4.2765
	#42	9829	4.1621
	#43	9840	4.1740
	#44	9828	4.1740
	#45	9818	4.1510
His	#1	12177	4.6080
	#2	12452	4.6076
	#3	11407	4.5380
	#4	11863	4.6085
	#5	11709	4.6269
	#6	11729	4.6300
	#7	11726	4.6371
	#8	12046	4.5872
	#9	12470	4.6030
	#10	13852	4.6351
	#11	16027	4.6090
	#12	11835	4.6633
	#13	11986	4.6947
	#14	11996	4.6696
	#15	11690	4.6490
	#16	12026	4.6633
	#17	12436	4.6803
	#18	12161	4.7000
	#19	12736	4.6592
	#20	11784	4.6820
	#21	11577	4.5689
	#22	11544	4.5114
	#23	11626	4.5511

	#24	11533	4.5265
	#25	11726	4.5776
	#26	12083	4.5996
	#27	12955	4.5910
	#28	15078	4.5850
	#29	11614	4.6358
	#30	11669	4.7087
	#31	11606	4.7335
	#32	11667	4.6729
	#33	12042	4.6582
	#34	12882	4.6200
	#35	12401	4.6275
	#36	11739	4.6815
	#37	11626	4.6575
	#38	11863	4.7074
	#39	12236	4.6758
	#40	13216	4.7570
	#41	12450	4.7360
	#42	11561	4.5658
	#43	11573	4.5560
	#44	11562	4.5590
	#45	11551	4.5750
Ser (AGY)	#1	12246	3.9432
	#2	12516	3.9226
	#3	11470	3.8230
	#4	11937	3.9673
	#5	11786	4.0058
	#6	11800	3.9198
	#7	11797	4.0196
	#8	12115	3.9224
	#9	12539	3.9566
	#10	13916	3.8990
	#11	16091	3.8540
	#12	11898	3.9475
	#13	12055	3.9418
	#14	12059	4.0583
	#15	11755	4.0860
	#16	12092	4.0226
	#17	12504	3.9476
	#18	12228	3.9595
	#19	12805	3.9348
	#20	11855	3.9390

	#21	11647	3.8832
	#22	11615	3.8231
	#23	11698	3.9336
	#24	11609	3.8605
	#25	11795	4.0124
	#26	12153	3.9631
	#27	13025	3.7792
	#28	15146	3.7375
	#29	11684	3.9530
	#30	11739	3.9573
	#31	11675	3.9375
	#32	11736	3.8848
	#33	12111	3.7779
	#34	12949	3.6962
	#35	12469	3.7835
	#36	11809	3.9791
	#37	11696	3.9220
	#38	11933	4.0408
	#39	12306	4.0372
	#40	13285	4.0278
	#41	12519	4.0860
	#42	11631	3.9488
	#43	11642	3.9678
	#44	11632	4.0515
	#45	11620	3.9310
Glu	#1	3133	4.5367
	#2	5805	4.5084
	#3	1321	4.4715
	#4	1526	4.5696
	#5	2111	4.5624
	#6	1332	4.6091
	#7	1293	4.6007
	#8	1046	4.5780
	#9	1122	4.6202
	#10	869	4.5939
	#11	1167	4.6095
	#12	632	4.5770
	#13	199	4.5154
	#14	119	4.5408
	#15	37	4.5375
	#16	188	4.5170
	#17	1275	4.4927

	#18	1256	4.4910
	#19	1295	4.4944
	#20	1289	4.5895
	#21	965	4.5296
	#22	1337	4.5639
	#23	1376	4.5442
	#24	1466	4.5485
	#25	1284	4.5397
	#26	1247	4.4825
	#27	1152	4.4018
	#28	1162	4.3940
	#29	661	4.4898
	#30	332	4.4606
	#31	1344	4.4285
	#32	1464	4.4245
	#33	1267	4.4932
	#34	1233	4.4998
	#35	1212	4.5160
	#36	71	4.4958
	#37	1292	4.4770
	#38	3	4.5160
	#39	20	4.4724
	#40	2334	4.5018
	#41	126	4.4715
	#42	1282	4.5178
	#43	1282	4.5432
	#44	1283	4.5435
	#45	1283	4.4940
Thr	#1	671	5.6749
	#2	47	5.6356
	#3	15161	5.5030
	#4	14913	5.7207
	#5	15488	5.6657
	#6	15507	5.6652
	#7	15508	5.6340
	#8	14273	5.7847
	#9	14102	5.7514
	#10	11764	5.7041
	#11	9054	5.6780
	#12	15604	5.7322
	#13	15764	5.7683
	#14	15416	5.6980

#15	15475	5.6770
#16	15818	5.6871
#17	16234	5.6990
#18	15939	5.7115
#19	16554	5.6856
#20	15576	5.6490
#21	14471	5.8228
#22	13799	5.8060
#23	11640	5.6727
#24	15253	5.6255
#25	8697	5.7235
#26	3507	5.7179
#27	332	5.6682
#28	3093	5.6970
#29	15293	5.8423
#30	15277	5.9237
#31	15335	5.9784
#32	15408	5.9903
#33	15776	6.0085
#34	16558	6.0078
#35	16059	6.0180
#36	15213	5.8636
#37	15375	5.8900
#38	15040	5.8352
#39	15421	5.8031
#40	16399	5.9152
#41	15631	5.9050
#42	15308	5.7641
#43	15318	5.7386
#44	15314	5.7710
#45	15298	5.7880

[a] Positions of the nodes in a composite tree of the sampled species were shown in supplementary Fig. S2.

Table S4 - Correlation coefficients and slope of the linear regression line between tRNA position (base pair distance from the 3´-end of control region) and usage of codons corresponding to each tRNA (logarithm of the absolute number) in the evolutionarily-stable mitochondrial gene order and 17 representative mitochondrial gene orders with rearranged tRNA positions

Scientific name	Common name	Accession No.	Slope [a]	r [b]	n	p [c]
Evolutionarily stable gene order (average of 33 species)			-2.3×10^{-5}	–0.1440	22	0.2613
Rearranged gene orders within lower taxa						
Actinopterygii						
Eurypharynx pelecanoides	Pelican eel	AB046473	**-5.2×10^{-5}**	–0.3804	22	0.0404
Saccopharynx lavenbergi	Gulper eel	AB047825	**-4.8×10^{-5}**	–0.3758	22	0.0424
Gonostoma gracile	Slender fangjaw	AB016274	-0.3×10^{-5}	–0.0168	22	0.4703
Myctophum affine	Lantern fish	AP002922	-0.7×10^{-5}	–0.0453	22	0.4207
Caelorinchus kishinouyei	Grenadier	AP002929	-1.8×10^{-5}	–0.1268	22	0.2870
Aspasma minima	Clingfish	AP004453	-1.2×10^{-5}	–0.0920	22	0.3420
Aulostomus chinensis	Trumpet fish	AP009197	-0.3×10^{-5}	–0.0202	22	0.4644
Chlorurus sordidus	Parrot fish	AP006567	-1.3×10^{-5}	–0.0817	22	0.3589
Amphibia						
Rana nigromaculata	Pond frog	AB043889	**-6.4×10^{-5}**	–0.4131	22	0.0280
Buergeria buergeri	Kajika frog	AB127977	**-8.2×10^{-5}**	–0.4663	22	0.0143
Rhacophorus schlegelii	Tree frog	AB202078	**-6.2×10^{-5}**	–0.3632	22	0.0483
Reptilia						
Boa constrictor	Boa	AB177354	-1.7×10^{-5}	–0.1086	22	0.3153
Dinodon semicarinatus	Colubrid snake	AB008539	-0.8×10^{-5}	–0.0491	22	0.4142
Gloydius blomhoffii	Pit viper	EU913477	0.5×10^{-5}	0.0263	22	0.4538
Sphenodon punctatus	Tuatara	AF534390	**-7.4×10^{-5}**	–0.3562	20	0.0616
Crocodylus niloticus	Nile crocodile	AJ810452	0.6×10^{-5}	0.0408	22	0.4285
Varanus komodoensis	Komodo dragon	AB080275 [d] AB080276 [d]	-0.3×10^{-5}	–0.0172	22	0.4697

[a] Slopes of the linear regression line. Bold letters indicate the slopes which seem to show stronger relationship than that in the evolutionarily stable gene order

[b] Pearson’s correlation coefficients

[c] One-tailed p values of the correlation coefficients

[d] Mitochondrial genome sequence of Komodo dragon is divided into two GenBank entries
